# Gingerol Synergizes the Cytotoxic Effects of Doxorubicin against Liver Cancer Cells and Protects from Its Vascular Toxicity

**DOI:** 10.3390/molecules21070886

**Published:** 2016-07-08

**Authors:** Fahad A. Al-Abbasi, Eman A. Alghamdi, Mohammed A. Baghdadi, Abdulmohsin J. Alamoudi, Ali M. El-Halawany, Hany M. El-Bassossy, Ali H. Aseeri, Ahmed M. Al-Abd

**Affiliations:** 1 Department of Biochemistry, Faculty of Science, King Abdulaziz University, Jeddah 21523, Saudi Arabia; alabassif@hotmail.com (F.A.A.-A.); mo0on_mo0ony@yahoo.com (E.A.A.); m-baghdadi@hotmail.com (M.A.B.); 2 Research Centre, King Faisal Specialist Hospital and Research Centre, Jeddah 21499, Saudi Arabia; 3 Department of Pharmacology and Toxicology, Faculty of Pharmacy, King Abdulaziz University, Jeddah 21589, Saudi Arabia; abdulmohsin@outlook.com (A.J.A.); helbassossy@kau.edu.sa (H.M.E.-B.); 4 Department of Natural Products and Alternative Medicine, Faculty of Pharmacy, King Abdulaziz University, Jeddah 21589, Saudi Arabia; ahalawany2003@yahoo.com; 5 Pharmacognosy Department, Faculty of Pharmacy, Cairo University, Cairo 11562, Egypt; 6 Faculty of Pharmacy, Zagazig University, Zagazig 44519, Egypt; 7 Ministry of Health, Jeddah 21484, Saudi Arabia; alhaseeri@moh.gov.sa; 8 Department of Pharmacology, Medical Division, National Research Centre, Giza 12622, Egypt

**Keywords:** hydroxyphenylalkanes, diarylheptanoids, gingerol, doxorubicin, liver cancer, vascular protection

## Abstract

Hydroxyphenylalkanes and diarylheptanoids possess potential therapeutic value in different pathophysiological conditions, such as malignancy. In the current study, naturally isolated hydroxyphenylalkane and diarylheptanoid compounds were investigated for potential chemo-modulatory effects in addition to potential vascular protective roles with doxorubicin. Diarylheptanoids showed stronger antioxidant effects, in comparison to hydroxyphenylalkanes, as demonstrated by DPPH assay and amelioration of CCl_4_-induced disturbed intracellular GSH/GSSG balance. Shogaol and 4′-methoxygingerol showed considerable cytotoxic effects against HCT116, HeLa, HepG2 and MCF7 cells, with IC_50_ values ranging from 3.1 to 19.4 µM. Gingerol significantly enhanced the cytotoxic profile of doxorubicin against HepG_2_ and Huh7, cells decreasing its IC_50_s by 10- and 4-fold, respectively. Cell cycle distribution was studied using DNA cytometry. Doxorubicin alone induced cell accumulation at S-phase and G_2_/M-phase, while in combination with gingerol it significantly induced cell cycle arrest at the G_2_/M-phase. Additionally, the vascular protective effect of gingerol against doxorubicin (10 µM) was examined on isolated aortic rings. Co-incubation with 6-gingerol (30 µM) completely blocked the exaggerated vasoconstriction and impaired vascular relaxation induced by doxorubicin. In conclusion, despite its relatively weak antioxidant properties, gingerol protected from DOX-induced vascular damage, apparently not through a ROS scavenging mechanism. Besides, gingerol synergized the cytotoxic effects of DOX against liver cancer cells without influencing the cellular pharmacokinetics.

## 1. Introduction

Grain of Paradise (*Aframomum melegueta* K. Schum, Zingiberaceae) is the only spice native to Africa and considered as an African panacea [1]. Seeds of *A. melegueta* were used, as a folk remedy, for the treatment of diarrhoea, and painful inflammatory conditions and in the control of postpartum haemorrhages [2]. Anti-ulcer, cytoprotective, antimicrobial, anti-nociceptive and aphrodisiac effects of the aqueous seed extract are also reported [3,4]. Phytochemical investigations of the plant seeds revealed the presence of paradol- and gingerol-like compounds, in addition to diarylheptanoids with hepatoprotective and estrogenic effects [5,6].

6-Gingerol is a major hydroxyphenylalkane isolated from *A. melegueta* and present in several plants belonging to the family Zingiberaceae, such as ginger and cardamom. The formerly mentioned plants are widely used in the Middle Eastern and Asian cuisine as a spice and everyday beverage. 6-Gingerol is reported to display several biochemical and pharmacological activities, such as cancer chemopreventive, anti-mutagenic, anti-apoptotic [7], anti-oxidant, anti-inflammatory [8], cardio- and hepatoprotective effects [5,9]. Gingerol is also known to inhibit the enzymes nitric oxide synthase and cyclo-oxygenase [10] and to suppress the expression of tumor necrosis factor alpha (TNF-α) [11]. 6-Paradol, another major constituent of *A. melegueta*, is closely related in structure to gingerol with one hydroxyl less in the alkyl chain. It is reported to possess chemopreventive, antioxidant and anti-inflammatory effects [12]. Paradol and its derivatives induce apoptosis through a caspase-3-dependent mechanism [13]. In spite of its structure similarity to gingerol, paradol’s biological activity is less explored than that of gingerol.

Diarylheptanoids are chemically characterized by the presence of an aryl-C7-aryl moiety. Diarylheptanoids from *Pinus flexilis* (E. James) possess protein kinase C inhibitory effects [14]. In addition, a cytotoxic diarylheptanoid was isolated from the roots of *Juglans mandshurica* (Maxim.) [15]. Diarylheptanoids with a carbonyl group at C-3, isolated from bark of black alder are also reported to inhibit the growth of resistant lung carcinoma. The active compounds were found to increase doxorubicin accumulation in cancer cells through modulation of P-gp activity [16].

The burden of neoplasia is increasing globally, with several millions deaths per year. Liver malignancies are the second most prevalent type of solid tumor, with an annual mortality of half a million among males and a similar number among females [17]. Doxorubicin (DOX) is a cytotoxic anthracycline used successfully for the treatment of several malignancies, such as liver cancer [18,19,20]. A major limitation for DOX treatment and a major cause of course treatment non-compliance is its intolerable cardiovascular side effects [21,22]. Several antioxidants were reported to have protective effect against doxorubicin-induced cardiovascular toxicity [9,23]. However, negative influence of free radical scavenging state might ameliorate the primary DOX anticancer properties [24,25,26]. In our previous work, resveratrol and didox (powerful antioxidants) marginally potentiated the effect of DOX against liver cancer cells and protected from its cardiotoxicity [27,28]. Apart from its toxicity, the efficacy of DOX is greatly affected by overexpression of ATP-dependent efflux pump P-glycoprotein (P-gp) [29]. It was reported previously that hydroxyphenylalkanes and diarylheptanoids are potential P-gp efflux pump inhibitors and hence might potentiate the activity of several P-gp substrates such as DOX [30]. In the current work, we isolated several naturally occurring hydroxyphenylalkanes and diarylheptanoids from *A. melegueta* K. Schum (Zingiberaceae). After rational preliminary biological screening of the isolated compounds, 6-gingerol was selected to protect from doxorubicin-induced vascular toxicity besides potentiating its anticancer properties against liver cancer cells. 

## 2. Results

### 2.1. Isolation and Structural Identification of Hydroxyphenylalkanes and Diarylheptanoids from A. melegueta

**Figure 1:**
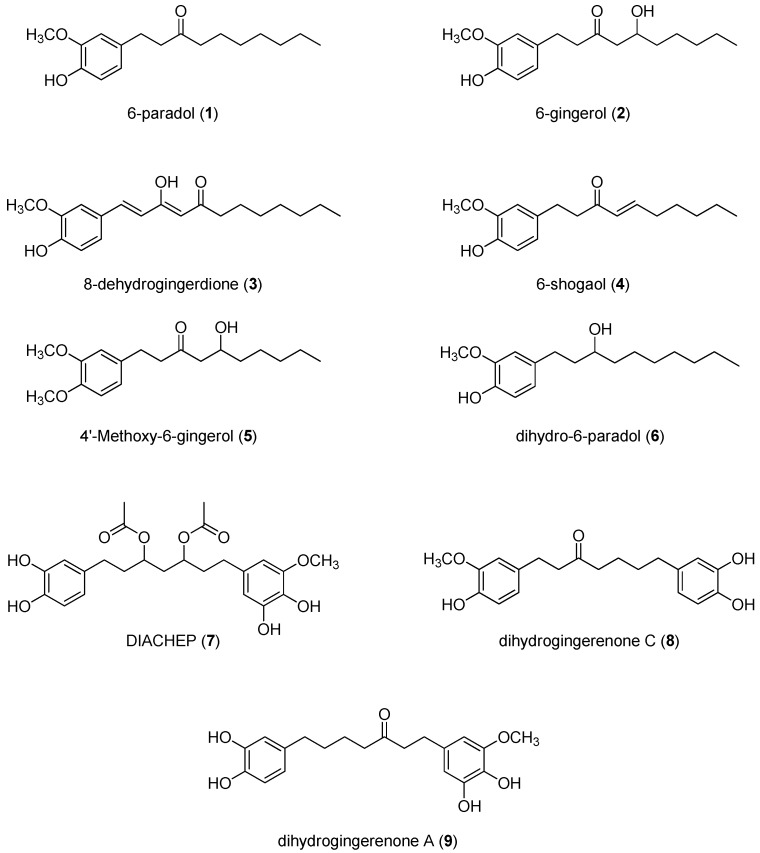
Compounds isolated from *Aframomum melegueta*.

The chloroform fraction of *A. melegueta* yielded three diarylheptanoids and six hydroxylphenyl-alkanes (Figure 1). The compounds were identified based on their ^1^H- and ^13^C-NMR data (see Supplementary Materials) and by comparison with reported literature as follows: 6-paradol (**1**) [31,32,33,34], 6-gingerol (**2**) [32], 8-dehydrogingerdione (**3**) [5], 6-shogaol (**4**) [33,34], 4′-methoxy-6-gingerol (**5**) [35], dihydro-6-paradol (**6**) [33], 3,5-diacetoxy-1-(3′,4′-dihydroxylphenyl)-7-(3″,4″-dihydroxy-5″-methoxyphenyl)heptane, DIACHEP (**7**) [31], dihydrogingerenone C (**8**) [6], and dihydrogingerenone A (**9**) [6].

### 2.2. Anti-Oxidant Activity of Isolated Hydroxyphenylalkanes and Diarylheptanoids

**Figure 2:**
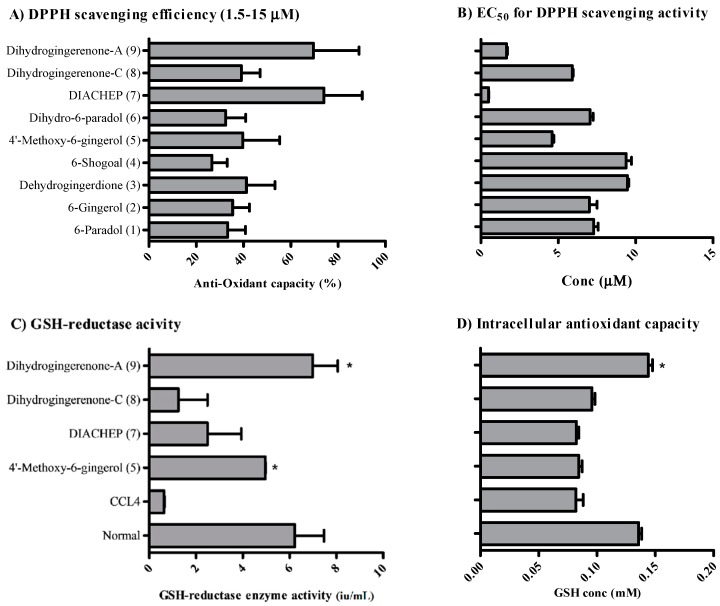
Antioxidant activity of isolated hydroxyphenylalkanes and diarylheptanoids was tested using a cell free system and with HepG_2_ cells. Antioxidant capacities of the test compounds were tested using the DPPH free radical scavenging assay; scavenging efficiency (**A**) and EC_50_s (**B**) were calculated over concentration range of 0.5 to 50 µM. GSH reductase enzyme activity (**C**) and GSH concentration (**D**) were determined in CCl_4_ challenged HepG_2_ cells. Data is presented as mean ± SD; *n* = 3. *: significantly different from CCl_4_ treated group.

Hydroxyphenylalkanes and diarylheptanoids are known for their robust antioxidant activity. We tested their free radical scavenging activity using the DPPH assay. DIACHEP (**7**), dihydrogingerenone A (**9**), dihydrogingerenone C (**8**) and 4′-methoxy-6-gingerol (**5**) showed the strongest free radical scavenging activity, with EC_50_s of less than 5 μM and average free radical scavenging efficiencies higher than 40% (Figure 2A,B). Potent free radical scavenging hydroxyphenylalkanes and diarylheptanoids were tested in CCl_4_-challenged HepG2 cells. CCl_4_ significantly abolished the GSH-reductase activity in HepG2 cells. Only dihydrogingerenone A (**9**) and 4′-methoxy-6-gingerol (**5**) reversed CCl_4_-induced GSH reductase enzyme activity reduction (Figure 2C). Additionally, CCl_4_ significantly decreased the reduced GSH concentration; only dihydrogingerenone A (**9**) reversed CCl_4_-induced GSH reduction (Figure 2D). None of the hydroxyphenylalkanes and diarylheptanoids under investigation exerted any significant effect against GSH peroxidase enzyme (data not shown).

### 2.3. Cytotoxicity Assessment of Hydroxyphenylalkanes and Diarylheptanoids

The SRB-U assay was used to assess the cytotoxicity of nine naturally occurring hydroxyphenylalkanes and diarylheptanoids against four different tumor cell lines over a concentration range of 0.01–100 μM. The tested compounds showed variable cytotoxicities against the cell lines under investigation (HCT-116, HepG2, MCF-7 and HeLa cell lines). However, HepG2 cells were relatively more resistant, while HCT-116 was the most sensitive cell line.

In HCT-116 colorectal cancer cells, all tested compounds showed considerable cytotoxicities, with IC_50_s ranging from 1.5 μM to 12.6 μM. In addition, resistant fraction (R)-values for all hydroxyphenylalkanes and diarylheptanoids under investigation (except gingerol) were less than 15%. It is worth mentioning that, despite the relatively low IC_50_ of gingerol (1.5 μM), it suffered from considerably high resistance (60.6%) within HCT-116 cells (Table 1).

The HepG2 liver cancer cell line possessed the highest resistance to hydroxyphenylalkanes and diarylheptanoids amongst other tumor cell types. In HepG2 cells, only dihydro-6-paradol (**6**), 6-shogoal (**4**) and 4′-methoxy-6-gingerol (**5**) showed relatively potent cytotoxicity with IC_50_s of 13.5 μM, 18.7 μM and 19.4 μM, respectively. However, dihydro-6-paradol (**6**) suffered from up to 50.2% resistant fraction. Gingerol (**2**) showed moderate cytotoxicity against HepG2 cells with an IC_50_ of 71.9 μM. The rest of tested compounds did not show any notable cytotoxicity, with IC_50_s higher than 100 μM (Table 1).

With respect to MCF-7 breast cancer cells, paradol (**1**), shogoal (**4**), dihydrogingerenone C (D 11) and 4′-methoxygingerol (**5**) showed considerable cytotoxicities, with IC_50_s of 22.0, 20.4, 7.5, 20.4 and 12.0 μM, respectively. However, the resistant fractions for paradol (**1**) and dihydrogingerenone C (**8**) were higher than 40%. DIACHEP (**7**), dihydro-6-paradol (**6**), dihydrogingerenone-A (**9**) and dehydrogingerdione (**3**) showed moderate cytotoxicities, with IC_50_s ranging from 59.4 μM to 63.7 μM. Dihydro-6-paradol (**6**) also suffered from resistance up to 42.9% in MCF-7 cells. Finally, gingerol showed weak cytotoxicity, with an IC_50_ higher than 100 μM (Table 1).

Like HCT-116, the majority of hydroxyphenylalkanes and diarylheptanoids under investigation showed considerable cell killing effect toward HeLa cells. DIACHEP (**7**), 6-gingerol (**2**), dihydro-6-paradol (**6**), 6-shogoal (**4**), dihydrogingerenone-A (**9**), dihydrogingerenone C (**8**) and 4′-methoxy-6-gingerol (**5**) killed HeLa cells with IC_50_s equal to or less than 20 μM. Paradol (**1**) and dehydrogingerdione (**3**) were relatively weaker against HeLa cells with IC_50_s of 57.7, 27.6 and 55.8 μM, respectively. HeLa did not show considerable resistance to the majority of hydroxyphenylalkanes and diarylheptanoids under investigation, with R-values equal to or less than 15%. Only 6-gingerol (**2**) and 6-paradol (**1**) suffered from considerable resistance, with R-values of 43.8% and 37.9%, respectively (Table 1).

### 2.4. The Influence of Hydroxyphenylalkanes and Diarylheptanoids on the Cellular Pharmacokinetics

**Figure 3:**
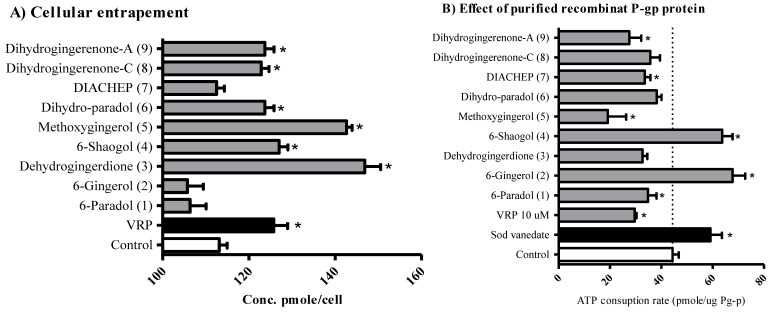
The effect of isolated compounds on the activity of P-glycoprotein efflux pump within CaCo-2 cells (**A**) and in cell free isolated recombinant P-gp protein (**B**). *: significantly different from control group.

Multidrug resistance in particular tumor types, such as solid tumors within the gastrointestinal tract, is highly attributed to impaired cellular pharmacokinetics and intracellular drug entrapment issues. The ability of hydroxyphenylalkanes and diarylheptanoids to enhance the cellular entrapment of P-glycoprotein substrates was tested within CaCo-2 colorectal cancer cells. Dihydro-6-paradol (**6**), 6-shogoal (**4**), dihydrogingerenone-A (**9**), dihydrogingerenone C (**8**), and 4′-methoxy-6-gingerol (**5**) significantly increased the cellular entrapment of doxorubicin (P-gp probe) and increased its intracellular concentration from 113.1 ± 2.6 pmole/cell to 123.7 ± 3.0 pmole/cell, 127.0 ± 2.8 pmole/cell, 123.71 ± 3.0 pmole/cell, 122.9 ± 2.5 pmole/cell, 146.9 ± 5.2 pmole/cell and 142.6 ± 1.9 pmole/cell, respectively (Figure 3A).

Further investigation for the sub-molecular interaction between the isolated compounds and P-gp subunits was undertaken using human recombinant P-gp membrane bound protein linked to ATPase enzyme subunit. Competitive P-gp inhibitors such as verapamil (VRP) are supposed to increase ATPase activity due to conformational changes and results in more ATP consumption (33.3% less remaining ATP concentration compared to basal ATP consumption). On the other hand, direct ATPase inhibitors such as sodium vanadate would decrease ATP consumption (133.1% more remaining ATP concentration compared to basal consumption condition). Only 6-shogoal (**4**) and 6-gingerol (**2**) showed pure ATPase inhibitory effects with 143.5% and 152.7% remaining ATP concentration, respectively (Figure 3B). On the other hand, DIACHEP (**7**), 6-paradol (**1**), dihydrogingerenone-A (**9**) and 4′-methoxy-6-gingerol (**5**) significantly decreased ATP remaining concentration to 75.5%, 78.2%, 50.2%, 61.9% and 43.2% of control level. This is indicative of increased ATP consumption and could be attributed to competitive P-gp inhibition (Figure 3B). Other hydroxyphenylalkanes and diarylheptanoids under investigation did not induce any significant change for ATP consumption rate. This might be attributed to lack of interaction with either subunit of P-gp molecules or to dual interaction with both subunits, yet dihydro-6-paradol (**6**), dihydrogingerenone C (**8**) and dehydrogingerdione (**3**) significantly increased cellular entrapment of P-gp probe. Accordingly, it is suggested that these compounds interact with both subunits of P-gp molecules. On the other hand, DIACHEP (**7**), and 6-paradol (**1**) did not exert any significant interaction with either subunit of P-gp molecules.

### 2.5. Chemomodulatory Effect of Gingerol on Doxorubicin within Liver Cancer Cells

**Figure 4:**
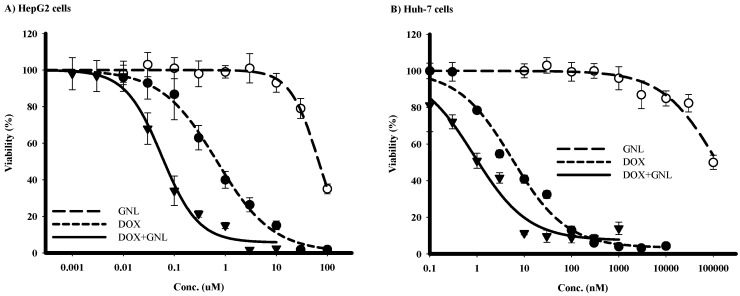
The effect of 6-gingerol (GNL) on the cytotoxicity of DOX in HepG2 (**A**) and Huh-7 (**B**) cell lines. Cells were exposed to serial dilution of DOX (●), GNL (○) or their combination (▼) for 72 h. Cell viability was determined using SRB assay.

DOX exerts its cell killing effects via the generation of intracellular free radicals. Based on its moderate antioxidant activity (Figure 2), and moderate cytotoxic effects (Table 1), gingerol would be a good candidate to improve the activity of doxorubicin against liver cancer cells. Huh-7 cells were far more sensitive to DOX, compared to HepG_2_, with IC_50_ of 4.6 ± 0.9 nM and 680 ± 60 nM, respectively. Gingerol exerts weaker cytotoxic effects against Huh-7 cells compared to HepG_2_ cells with IC_50_’s of 103.1 ± 3.0 µM and 71.9 ± 2.8 µM, respectively (Figure 4A,B and Table 2). However, 6-gingerol synergistically improved the cytotoxic profile of DOX against both of these liver cancer cell lines. 6-Gingerol significantly decreased the IC_50_’s of DOX form 680 ± 60 nM and 4.6 ± 0.9 nM to 67.4 ± 9.1 nM and 1.2 ± 0.13 nM in HepG_2_ and Huh-7 cells, respectively. The combination indices for equitoxic combination of gingerol and DOX within HepG_2_ and Huh-7 cells were 0.19 and 0.29, respectively (Table 2). It is worth mentioning that, gingerol combination with DOX did not influence the resistance fraction to DOX within either HepG_2_ or Huh-7 cells.

### 2.6. Cell Cycle Distribution Analysis of Liver Cancer Cells

**Figure 5:**
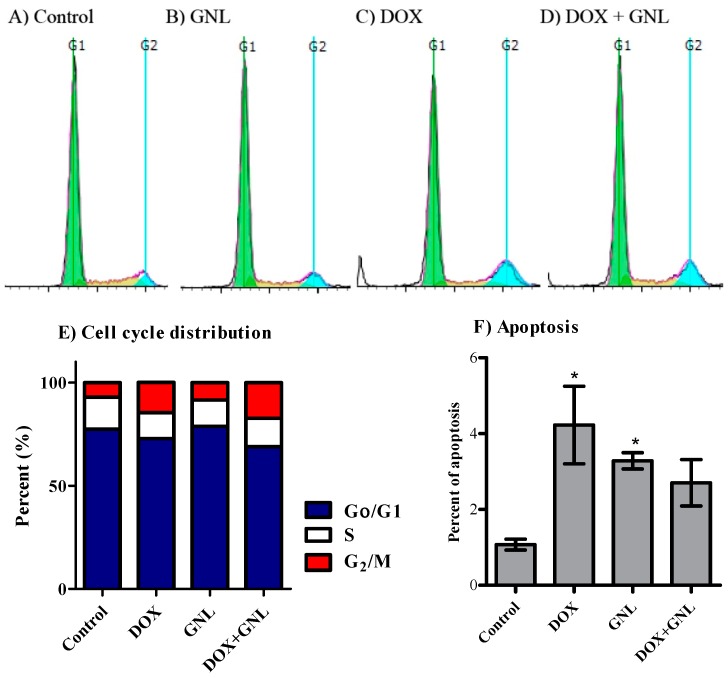
Effect of 6-gingerol (GNL) on the cell cycle distribution of HepG2 cells. The cells were exposed to GNL (**B**); DOX (**C**); or combination of GNL and DOX (**D**) for 24 h and compared to control cells (**A**); Cell cycle distribution was determined using DNA cytometry analysis and different cell phases were plotted (**E**) as percentage of total events; Sub-G cell population was taken as representative of late apoptosis/necrosis and was plotted as percent of total events (**F**). Data is presented as mean ± SD; *n* = 3. *: significantly different from control group.

Cell cycle distribution determined using DNA flow cytometry was used to investigate the nature of the interaction between DOX and gingerol. In HepG_2_ cells, gingerol significantly decreased the cell population is S-phase from 15.5% ± 0.7% to 12.7% ± 1.3%. DOX treatment alone significantly increased the cell population in G_2_/M phase (from 7.1% ± 0.7% to 14.6% ± 3.1%) with a reciprocal decrease in the S-phase cell population (from 15.5% ± 0.7% to 12.5% ± 1.2%) (Figure 5A–C,E). The combination of DOX with gingerol induced significant cell cycle arrest at the G_2_/M phase (17.4% ± 2.4%) compared to the control group (7.1% ± 0.7%). The induced G_2_/M cell phase arrest was accompanied by reciprocal decrease in the non-proliferating cell population (G_0_/G_1_-phase) from 77.4% ± 1.0% to 68.9% ± 3.0% (Figure 5A,D,E). 

Finally, exposure of HepG2 cells to DOX or gingerol alone for 24 h resulted in significant cell death observed by elevated pre-G cell population form 1.1% ± 0.2% to 4.2% ± 1.0% and 3.3% ± 0.2%, respectively. Gingerol combination with DOX did not inhibit DOX-induced cell kill, where no significant change in pre-G cell population was observed after combination treatment compared to cells treated with DOX alone (Figure 5F).

**Figure 6:**
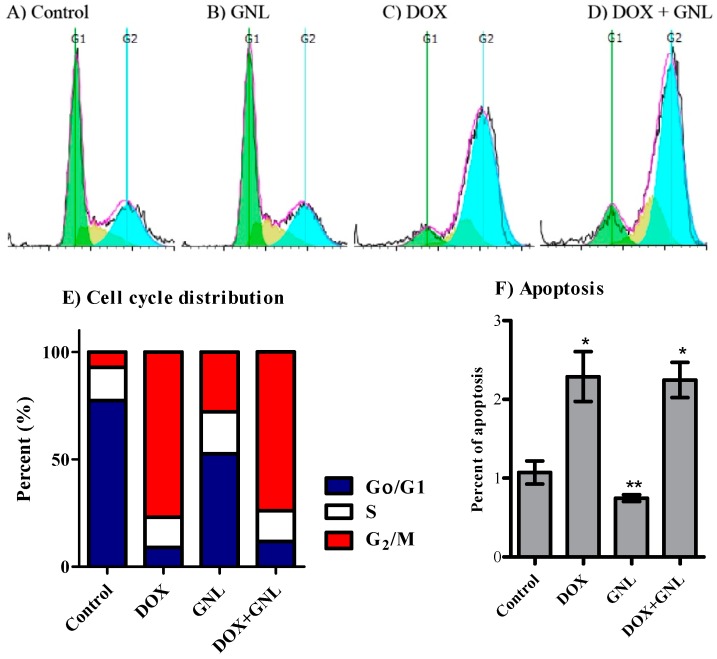
Effect of GNL on the cell cycle distribution of Huh-7 cells. The cells were exposed to GNL (**B**); DOX (**C**); or combination of GNL and DOX (**D**) for 24 h and compared to control cells (**A**); Cell cycle distribution was determined using DNA cytometry analysis and different cell phases were plotted (**E**) as percentage of total events; Sub-G cell population was taken as representative of late apoptosis/necrosis and was plotted as percent of total events (**F**). Data is presented as mean ± SD; *n* = 3. *: significantly different from control group; **: significantly different from DOX group.

With respect to Huh-7 cells, gingerol significantly decreased the cell population in G_0_/G_1_-phase (from 77.4% ± 1.0% to 52.6% ± 2.0%) with a reciprocal increase in both S-phase (from 15.5% ± 0.4% to 19.6% ± 1.2%) and inG_2_/M phase (from 7.1% ± 0.7% to 27.8% ± 3.2%) cell populations (Figure 6A,B,E). DOX alone significantly arrested cells at G_2_/M-phase (77.0% ± 2.0%) compared to control (7.1% ± 0.7%); with reciprocal abolishment for cells in G_0_/G1-phase from 77.4% ± 1.0% to 8.9% ± 0.1% (Figure 6A,C,E). The combination of gingerol with DOX similarly arrested cells at G_2_/M-phase (76.2% ± 0.9%) compared to control (7.1% ± 0.7%); with reciprocal abolishment for cells in G_0_/G1-phase from 77.4% ± 1.0% to 11.8% ± 1.0% (Figure 6A,D,E). Finally, exposure of Huh-7 cells to DOX as well as combination of DOX with gingerol for 24 h resulted in significant cell death observed by an elevation of the pre-G cell population from 1.1% ± 0.2% to 2.3% ± 0.3% and 2.2% ± 0.2%, respectively (Figure 6F).

### 2.7. Gingerol Protects from DOX Induced Vascular Toxicity

**Figure 7:**
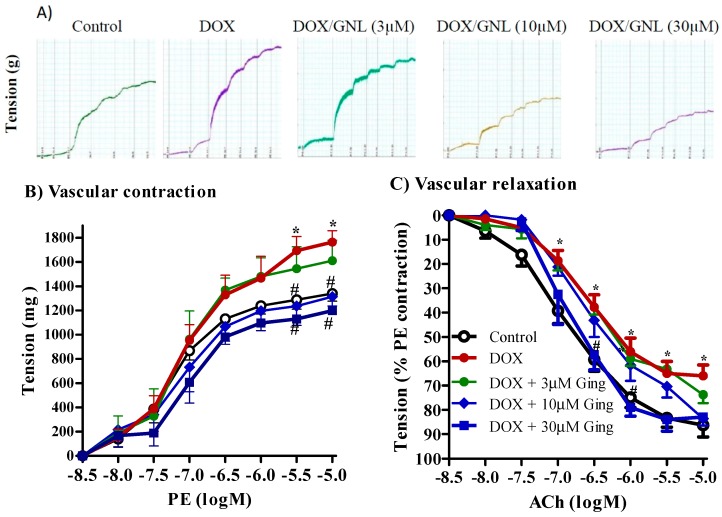
Effect of in vitro incubation with 6-gingerol (3, 10 and 30 µM) on doxorubicin (DOX, 10 µM)-induced exaggerated vasoconstriction to phenylephrine (PE), (**A**,**B**) and impaired vasodilation to acetylcholine (ACh) (**C**). A Data is presented as mean ± SD; *n* = 6–8. *: significantly different from control group; #: significantly different from DOX treated group.

The current study showed that aortae isolated from normal animals exposed to doxorubicin (10 µM, 1 h) exhibited exaggerated vasoconstriction in response to PE (10^−9^ to 10^−5^ M), compared to unexposed aortae. This enhancement of vasoconstriction was statistically significant (*p* < 0.05) at PE concentrations of 3 × 10^−5^ and 10^−5^ M (Figure 7B). Thirty minutes of incubation with 6-gingerol (0.3–30 µM) alleviated this DOX-induced exaggerated vasoconstriction of aortae in a concentration-dependent manner. The inhibition of PE (10^−5^ M)-induced contraction was significant at both tested concentrations of 6-gingerol (10 and 30 μM) with PE concentrations of 3 × 10^−5.5^ and 10^−5^ M. The highest concentration of 6-gingerol (30 μM) normalized the exaggerated contraction response to PE back to normal control value (Figure 7B).

Aortae exposed to DOX showed impaired vasodilation compared to unexposed aortae and this impaired relaxation was significant at ACh concentrations of 10^−7^ to 10^−5^ M. This impaired relaxation was alleviated by incubation with 6-gingerol in a concentration dependent manner and this alleviation was significant at the 30 μM concentration of 6-gingerol tested with PE concentrations of 3 × 10^−6.5^ and 10^−6^ M (Figure 7B).

## 3. Discussion

Natural products are eternal sources of active compounds. The therapeutic use of natural products has escalated from folk use of the whole plant or unprocessed natural entities [36] to the use of standardized extracts [37], followed by the isolation of specified compounds with defined molecular structures [38]. Grouping compounds of similar molecular structure sharing close pharmacological activity could shed light on structure-activity relationships and stimulate chemists to initiate the lead optimization process [39,40]. Herein, we isolated several structurally related hydroxyphenylalkane and diarylheptanoid compounds and assessed their potential cytotoxic and chemomodulatory effects, in addition to confirming their documented antioxidant activity.

All hydroxyphenylalkanes and diarylheptanoids under investigation showed considerable antioxidant activity in cell free systems and within HepG_2_ cells. However, diarylheptanoids could be considered more potent than hydroxyphenylalkanes in free radical scavenging capacity, as well as in restoring cellular GSH/GSSG balance. This might be attributed to the occurrence of an extra aromatic system in diarylheptanoids [41,42]. Some compounds such as DIACHEP (**7**) and dihydrogingerenone C (**8**) possessed free radical scavenging activity but failed to restore cellular GSH balance. This might be attributed to their slow cellular internalization [43]. Gingerol possessed moderate to weak antioxidant capacity compared to the rest of hydroxyphenylalkanes and diarylheptanoids. Our previous study showed that the vascular protective effects of gingerol might be attributed to direct vasodilatation and nitric oxide generation rather than free radical scavenging mechanism [44]. Besides, gingerol is reported as cardioprotective agent; and particularly against DOX-induced cardiac damage [9,45].

Hydroxyphenylalkanes and diarylheptanoids showed moderately potent cytotoxicity in the current work, with IC_50_s above 1 µM in all tested cell lines. However, these compounds showed preferable cytotoxicity against HCT-116 colorectal cancer cells. This might be partly attributed to the P-gp inhibitory effect of this group of compounds which could have induced excessive intracellular accumulation and auto-enhancement effects [46,47]. Although, P-gp is expressed in almost all gastrointestinal-related tumors [48,49], similar efficacies were not found against HepG_2_ liver cancer cells. This might be attributed to the mutated form of P-gp protein expressed in liver cancer [50].

The ability of hydroxyphenylalkanes and diarylheptanoids to interrupt the function of P-gp was tested functionally and at the sub-molecular level. Most of compounds under investigation improved the cellular pharmacokinetics of P-gp probe, while only 6-shogoal and 6-gingerol specifically inhibited the P-gp ATPase subunit. Other hydroxyphenylalkanes and diarylheptanoids are expected to inhibit the P-gp efflux pump via non-specific competitive binding or mixed ATPase inhibition/competitive binding. It would be better in terms of structure-activity relationship and lead optimization to design specific P-gp ATP-ase inhibitors rather than non-specific competitive binding inhibitors [30]. Interestingly, gingerol did not enhance the cellular entrapment of DOX within CaCo-2 cells. Besides the high expression of P-gp within CaCo-2 cells, it might be also explained by the abundance of other types of efflux proteins on the cell membrane of colorectal cancer cells such as MRP1, MRP2 or others [47].

Antioxidants might protect from the side effects and toxic manifestations of a wide variety of anticancer drugs such as doxorubicin [51,52]. However, substantial worries about the negative effect of these agents on the primary anticancer properties of chemotherapies cannot be discounted [26]. Herein and amongst this group of compounds, gingerol was selected for further investigation in the context of influencing the cytotoxicity of doxorubicin against liver cancer cells besides its protective effects against doxorubicin-induced vascular toxicity. Gingerol significantly synergized the cytotoxic effects of doxorubicin against two different liver cancer cells. In previous studies from our group different antioxidants (natural or synthetic) marginally enhanced the cytotoxic profile of doxorubicin, producing only additive drug interaction [27,28]. This might be supported by the weak antioxidant activity of gingerol. However, it cannot be attributed to enhanced cellular internalization of doxorubicin or cellular pharmacokinetic interaction. HepG2 cells also express different types of efflux pump proteins such as P-gp and BCRP [53] that might compensate for the specific P-gp ATPase inhibition activity of gingerol.

Pharmacodynamic interactions between doxorubicin and gingerol were studied using cell cycle distribution analysis. In Huh-7 cells, gingerol significantly induced cell accumulation in the S-phase as well as the G_2_/M-phase. Doxorubicin preferably intercalates with cellular DNA while cells are in either S-phase or G_2_/M-phase inducing cell cycle arrest at G_2_/M-phase, also called mitotic crises/catastrophe [54]. It was reported that agents inducing S-phase accumulation sensitize tumor cells to the killing effect of doxorubicin [55]. In contrast to Huh-7, HepG_2_ did not respond to gingerol treatment by S-phase accumulation; gingerol exerted clear antiproliferative effect accumulating cells at the G_0_/G_1_-phase. This explains the relatively weaker combined effect of gingerol and doxorubicin against HepG_2_ cells relative to Huh-7 cells. Luckily; gingerol did not hinder the activity of doxorubicin against HepG_2_ cells [56,57]. In other words, the synergism of gingerol with doxorubicin (CI-value = 0.19) against Huh-7 cells could be attributed to gingerol-induced cell cycle synchronization in the S-phase resulting in excessive sensitivity to doxorubicin. However, the weaker synergism (CI-value = 0.29) between gingerol and doxorubicin in HepG_2_ cells could be attributed to combined but independent antiproliferative and cytotoxic effects of gingerol and doxorubicin, respectively. Further molecular investigations to completely reveal the underlying possible pharmacodynamic interaction mechanisms between gingerol and doxorubicin in liver cancer cells are recommended.

Additionally, gingerol significantly protected against doxorubicin-induced vascular damage, in terms of restoring normal vascular contraction and relaxation. Our previous studies proved the protective effects of gingerol from doxorubicin induced cardiac damage at cardiomyocyte level [9]. Besides, we previously showed protective effects for gingerol against vascular complications of metabolic syndrome that was attributed to gingerol mediated vasodilatation [44]. In continuation of this research line, we presented experimental evidence for the functional protection of gingerol against doxorubicin induced vascular damage without ameliorating its inherent cytotoxicity against liver cancer cells.

## 4. Materials and Methods

### 4.1. Drugs and Chemicals

Verapamil (VRP), and Trypan Blue were purchased from Sigma Chemical Co. (St. Louis, MO, USA). Sulforhodamine-B (SRB) was purchased from Biotium Inc. (Hayward, CA, USA). Penicillin streptomycin and trypsin were purchased from Gibco (Grand Island, NY, USA). Phosphate buffer saline (PBS) was purchased from Becton Dickinson (Fullerton, CA, USA). RPMI-1640 media, DMEM media, fetal bovine serum (FBS), and other cell culture materials were purchased from ATCC (Houston, TX, USA). Other reagents were of the highest analytical grade.

### 4.2. General Experimental Procedures

Nuclear magnetic resonance (NMR, H, 400 MHz; ^13^C, 100 MHz) spectra were recorded on a JHA-LAA 400 WB-FT spectrometer (Jeol Co., Tokyo, Japan), the chemical shifts are presented as ppm with tetramethylsilane as an internal standard. TLC was carried out on pre-coated silica gel 60 F254 (0.25 mm, Merck; Darmstadt, Germany) and RP-18 F254S (0.25 mm, Merck Co.). Column chromatography (CC) was carried out on a BW-820MH silica gel, Wakosil C-300 silica gel (40–63 µm) (Wako Chem. Co., Osaka, Japan). Medium pressure liquid chromatography (MPLC) was performed on LiChroprep RP-18 (size A and B; Merck Co.).

### 4.3. Plant Material

Seeds of *A. melegueta* were purchased from the Harraz herbal store (Cairo, Egypt), and were identified by Assistant Prof. Dr. Sherif El-Khanagry, Agriculture Museum, El-Dokki, Cairo, Egypt. A voucher specimen has been kept in the herbarium of the Department of Pharmacognosy, Faculty of Pharmacy, Cairo University.

### 4.4. Extraction and Isolation of Compounds from A. melegueta

Seeds of *A. melegueta* (2.5 kg) were pulverized and extracted with MeOH (1 L) by cold maceration for three successive days. The pooled MeOH extracts were evaporated under vacuum to give a brown residue (130 g). The MeOH extract was suspended in water (500 mL) and fractionated using CHCl_3_ (1 L × 3) and the pooled fractions were evaporated under vacuum to yield chloroform fraction (90 g). The CHCl_3_ fraction was purified on silica gel column (70 cm × 5 cm) and eluted gradiently with hexane-EtOAc (5%–80%). The obtained fractions were pooled into ten sub-fractions 1–10.

Fraction 1 (15 g) was purified on a silica gel column (40 cm × 3 cm) eluted with hexane–EtOAc (9.5:0.5 *v*/*v*) to yield compound **1** (8 g). Fraction 3 (6.5 g) was chromatographed on a silica gel column (20 cm × 2.5 cm) and eluted with hexane–EtOAc (9:1 *v*/*v*) yielding **2** (2.5 g) and six subfractions. Sub-fraction 3–5 (1 g) was purified using a MPLC RP-18 column (size B) eluted with MeOH–H_2_O (8:2 *v*/*v*) to yield compounds **3** (4 mg), **4** (25 mg) and **5** (5 mg). Sub-fraction 3–8 (800 mg) was purified using a MPLC column size B eluted with MeOH–H_2_O (5:5–7:3 *v*/*v*) to obtain compound **6** (10 mg). Fraction 9 (13 mg) was chromatographed on a silica gel column (40 cm × 4 cm) and eluted with a hexane–EtOAc (9:1–5:5 *v*/*v*) gradient to yield sub-fractions 9-1–9-3. Sub-fraction 9-3 was purified on a silica gel column (25 cm × 2 cm) eluted with hexane–EtOAc (6:4 *v*/*v*) affording compounds **7** (1 g) and **8** (10 mg). The remainder of the fraction was applied to a MPLC column RP-18 (size A) eluted with MeOH–H_2_O (6:4 *v*/*v*) to afford compound **9** (20 mg).

### 4.5. Determining the Antioxidant Activity of Test Compounds Using Cell-Free System (DPPH Assay)

The antioxidant activity of the test compounds was evaluated using the DPPH radical scavenging method. Serial concentrations (0.5–50 µM) of test compounds were prepared with 0.4 mg/mL solution of DPPH in pure ethanol and left in the dark for 30 min. Absorbance at 520 nm was then measured and average free radical scavenging activity for each compound was calculated. Besides, EC_50_ were calculated from linear best fit regression analysis.

### 4.6. Cell Culture

Six different human solid tumor cell lines were used; colorectal cancer cell lines (HCT-116 and CaCo-2), cervical cancer cell line (HeLa), hepatocellular cancer cell lines (HepG2 and Huh-7), and breast cancer cell line (MCF-7). All Cell lines were obtained from VACSERA (Giza, Egypt). Cell lines were maintained in RPMI-1640 or DMEM media containing 100 U/mL penicillin; 100 µg/mL streptomycin, and supplemented with 10% heat-inactivated fetal bovine serum (FBS). Cells were propagated in a humidified cell culture incubator with 5% (*v*/*v*) CO_2_ at 37 °C.

### 4.7. Determining the Antioxidant Activity of Test Compounds within HepG2 Cells

To assess the potential free radical scavenging capacity of test compounds within the intracellular compartment, HepG_2_ cells (10^5^ cells) were challenged with CCl_4_ (40 mM) alone or with potentially active hydroxyphenylalkanes and diarylheptanoid compounds (5 µM) for 4 h. Media was collected and level of reduced glutathione (GSH) as well as the activity of glutathione reductase and peroxidase enzymes were measured as previously described [58,59,60].

### 4.8. Cytotoxicity Assessment

The cytotoxicity of the isolated compounds was tested against HCT-116, HeLa, HepG2, Huh-7 and MCF-7 cells by SRB assay as previously described [61]. Briefly, exponentially growing cells were collected using 0.25% Trypsin-EDTA and seeded in 96-well plates at 1000–2000 cells/well. Cells were treated with the isolated compounds for 72 h and subsequently fixed with TCA (10%) for 1 h at 4 °C. After several washings with water, cells were exposed to 0.4% SRB solution for 10 minutes at room temperature in dark place and subsequently washed with 1% glacial acetic acid. After the plates drying overnight, Tris-HCl was used to dissolve the SRB stained cells and color intensity was measured at 540 nm with ELISA microplate reader.

### 4.9. Data Analysis

The dose-response curves were analyzed as previously described [62] using the E_max_ model (Equation (1)):

(1) $$\%\text{ Cell viability}=\left(100-R\right)\times \left(1-\frac{{\left[D\right]}^{m}}{K_{d}{}^{m}+{\left[D\right]}^{m}}\right)+R$$

where R is the residual unaffected fraction (the resistance fraction), [D] is the drug concentration used, K_d_ or IC_50_ is the drug concentration that produces a 50% reduction of the maximum inhibition rate and m is a Hill-type coefficient. Absolute IC_50_ is defined as the drug concentration required to reduce absorbance by 50% of that of the control (i.e., K_d_ = absolute IC_50_ when R = 0 and E_max_ = 100 − R).

### 4.10. The Influence of the Naturally Hydroxyphenylalkanes and Diarylheptanoids on the Cellular Pharmacokinetics of Doxorubicin (DOX)

To assess the effect of hydroxyphenylalkanes and diarylheptanoids on cellular pharmacokinetics in colorectal cancer cells, their effect on the efflux pumping activity of P-gp was evaluated. Herein, doxorubicin (DOX) was used as P-gp fluorescent substrate. Intracellular DOX concentration was determined with and without co-exposure with hydroxyphenylalkanes and diarylheptanoids and compared to VRP as standard P-gp inhibitor (positive control). Briefly, exponentially proliferating CaCo-2 cells were plated in 6-well plates at plating density of 10^5^ cells/well. Cells were exposed to equimolar concentration of DOX (10 µM) and test compounds or VRP for 24 h at 37 °C and subsequently, extracellular DOX-containing media was washed three times in ice cold PBS. Intracellular DOX was extracted after cell lysis by sonication with saturated aqueous solution of ZnSO_4_ (100 µL), acetonitril (500 µL) and acetone (250 µL) for 20 min at 37 °C. After centrifugation, clear supernatant solution was collected and DOX concentration was measured spectroflourometrically at λ_ex/em_ of 482/550 nm. DOX concentration was normalized based on cell number [28].

### 4.11. Determining Sub-Molecular Interaction Characteristics between P-gp Protein and Naturally Occurring Hydroxyphenylalkanes and Diarylheptanoids

P-gp inhibitors block its efflux pumping activity via either competitive binding or inhibiting P-gp ATPase activity. Human recombinant membrane bound P-gp protein attached with ATPase subunit (Pgp-Glo™ Assay Systems, Promega Corporation, Madison, WI, USA) was used to determine the mechanism of P-gp inhibition via determining ATP consumption rate. Briefly, test compounds (10 µM) were incubated with Pgp-Glo™ assay systems according to manufacturer protocol. Rate of ATP consumption was calculated by measuring luminescent signal of the unmetabolized ATP via firefly luciferase system. Compound which covalent bind to P-gp molecule is supposed to stimulate ATPase subunit and increase ATP consumption; while ATPase inhibitor compounds would decrease ATPase subunit activity and decrease ATP consumption rate. Verapamil and sodium vanadate were used as positive controls (covalent binding and ATPase inhibitors, respectively). ATP consumption was expressed as remaining ATP concentration and normalized per P-gp protein concentration (pmole ATP/µg P-gp protein). 

### 4.12. Chemomodulatory Effect of Gingerol (GNL) to DOX within Liver Cancer Cells

Chemomodulatory effect of gingerol to doxorubicin (DOX) within liver cancer cells was determined using combination analysis between DOX and GNL as previously described [63]. Briefly, exponentially growing HepG2 and Huh-7 cells were seeded in 96-well plates (2000 cells/well) and exposed to equitoxic concentrations of DOX and GNL for 72 h. Cells were subsequently subjected to SRB assay as described in section 4.8. Combination index (CI-value) was calculated and used to define the nature of drug interaction (synergism if CI-value <0.8 as; antagonism if CI-value >1.2; and additive if CI-value ranges from 0.8–1.2). CI-value was calculated from the formula:

(2) $$\text{CI}-\text{value}=\frac{{\text{IC}}_{50}\text{drug}(x)\text{combination}}{{\text{IC}}_{50}\text{drug}(x)\text{alone}}+\frac{{\text{IC}}_{50}\text{drug}(y)\text{combination}}{{\text{IC}}_{50}\text{drug}(y)\text{alone}}$$

### 4.13. Analysis of Cell Cycle Distribution

To assess the effect of the doxorubcin, gingerol and their combination on cell cycle distribution, HepG2 and Huh-7 cells were treated with the pre-determined IC_50_s of both agents for 24 h. After treatment, cells were collected by trypsinization; washed twice with ice-cold PBS and re-suspended in 0.5 mL of PBS. Two milliliters of 70% ice-cold ethanol was added gently while vortexing. Cells were kept in ethanol solution at 4 °C for 1 h for fixation. Upon analysis, fixed cells were washed and re-suspended in 1 mL of PBS containing 50 μg/mL RNAase A and 10 μg/mL propidium iodide (PI). After 20 min incubation in dark place at room temperature, cells were analyzed for DNA contents by FACS-Vantage™ (Becton Dickinson Immunocytometry Systems). For each sample, 10,000 events were acquired. Cell cycle distribution was calculated using ACEA NovoExpress™ software (ACEA Biosciences Inc., San Diego, CA, USA).

### 4.14. Animals

Male Wistar rats (King Abdul-Aziz University, Jeddah, Saudi Arabia) weighing 120–140 g, aged 6 weeks were housed in clear polypropylene cages (three to four rats per cage) and kept under constant environmental conditions with equal light-dark cycle. Rats had free access to commercially available rodent pellet diet and purified water. All experimental procedures were performed in accordance with Saudi Arabia Research Bioethics and Regulations, which are consistent with the Guide for the Care and Use of Laboratory Animals published by the U.S. National Institutes of Health.

### 4.15. Assessing the Protective Effect of 6-gingerol Against Doxorubicin Induced Vascular Damage

To assess the protective effect of 6-gingerol on doxorubicin-induced vascular damage, deterioration in vascular reactivity was measured. Vascular reactivity of isolated thoracic aortae was determined using isolated artery techniques described in our previous publications [64,65]. Briefly, isolated aortae were co-incubated within organ bath with doxorubicin (10 µM) with or without different concentrations of 6-gingerol (0.3–30 µM) for 30 min before assessing the vasoconstriction and vasodilation responses compared to control aortic ring. For assessing the aortic contractile responsiveness, increases in tension due to cumulative additions of PE (10^−9^ to 10^−5^M) were recorded and expressed as milligram tension. In order to study the vasodilator responsiveness, aortic rings were first pre-contracted with maximal concentrations of PE (10^−5^M). Cumulative concentrations of ACh (10^−9^ to 10^−5^M) were then added to the organ bath and responses were recorded as percentage in relation to PE pre-contraction.

### 4.16. Statistical Analysis

Data are presented as mean ± SEM using GraphPad prism™ software (GraphPad Software Inc., La Jolla, CA, USA) for windows version 5.00. Analysis of variance (ANOVA) with Newman Keuls post hoc test was used for testing the significance using SPSS^®^ for windows, version 17.0.0. *p* < 0.05 was taken as a cut off value for significance.

## 5. Conclusions

In conclusion, gingerol, despite its relatively weak antioxidant properties, was found to protect from DOX-induced vascular damage, apparently not through its free radical scavenging mechanism. In addition, gingerol synergized the cytotoxic effects of DOX against liver cancer cells at pharmacodynamic level. The gingerol-induced chemomodulatory effect of doxorubicin was found to be independent on influencing P-gp efflux activity and cellular pharmacokinetics within liver cancer cells.

## Figures and Tables

**Table 1:** Cytotoxicity parameters of some naturally occurring hydroxyphenylalkanes and diarylheptanoids against different solid tumor cell lines.

Compound	HCT-116		HepG2		MCF-7		HeLa	
	IC_50_ (µM)	R-Value (%)	IC_50_ (µM)	R-Value (%)	IC_50_ (µM)	R-Value (%)	IC_50_ (µM)	R-Value (%)
6-Paradol (**1**)	10.4	15.1	>100	N/A	20.4	42.9	57.7	37.9
6-Gingerol (**2**)	1.5	60.6	71.9	N/A	>100	85.6	15.5	43.8
Dehydrogingerdione (**3**)	12.6	N/A	>100	N/A	63.7	N/A	55.8	1.1
6-Shogoal (**4**)	3.1	N/A	18.7	N/A	7.5	1.1	10.2	1.1
4′-Methoxy-6-gingerol (**5**)	11.4	4.6	19.4	N/A	12.0	N/A	9.2	0.5
Dihydro-6-paradol (**6**)	12.0	N/A	13.5	50.2	63.5	42.9	14.7	0.9
DIACHEP (**7**)	11.2	4.1	>100	N/A	61.5	N/A	20.2	0.94
Dihydrogingerenone-C (**8**)	10.5	10.7	N/A	75.5	20.4	65.5	16.6	15.1
Dihydrogingerenone-A (**9**)	12.2	7.8	N/A	N/A	59.4	0.7	17.5	5.5

**Table 2:** Effect of 6-gingerol (GNL) on the cytotoxicity parameters of DOX in liver cancer cells.

Treatment	*HepG_2_*		*Huh-7*	
	IC_50_	R-Value (%)	IC_50_	R-Value (%)
DOX	680 ± 60 nM	2.7 ± 0.8	4.6 ± 0.9 nM	3.0 ± 0.9
GNL	71.9 ± 2.8 µM	N/A	103.1 ± 3.0 µM	4.3 ± 1.7
DOX + GNL	67.4 ± 9.1 nM	3.3 ± 0.7	1.2 ± 0.13 nM	4.7 ± 1.1
CI-index/CI-value	Synergism/0.19		Synergism/0.29	

## Supplementary Materials

Supplementary materials can be accessed at: http://www.mdpi.com/1420-3049/21/7/886/s1.

## Abbreviations

The following abbreviations are used in this manuscript:

ACh	Acetylcholine
CCl_4_	Carbon tetrachloride
DIACHEP (**7**)	3,5-Diacetoxy-1-(3′,4′-dihydroxylphenyl)-7-(3″,4″-dihydroxy-5″-methoxyphenyl) heptane
DOX	Doxorubicin
GNL	Gingerol
GSH	Glutathione (reduced form)
GSSG	Glutathione (oxidized form)
iu	International Unit
P-gp	P-glycoprotein
PE	Phenylephrin
ROS	Reactive oxygen species
VRP	Verapamil
