# Supplementary material for: Gingerol Synergizes the Cytotoxic Effects of Doxorubicin against Liver Cancer Cells and Protects from Its Vascular Toxicity
Source: Molecules. 2016 Jul 8;21(7):886. doi: 10.3390/molecules21070886 (PMC6274287; doi:10.3390/molecules21070886)
Supplement: Supplementary file 1 [file molecules-21-00886-s001.pdf]

Supplementary Materials: Gingerol Synergizes the Cytotoxic Effects of Doxorubicin against Liver Cancer Cells and Protects from Its Vascular Toxicity

Fahad A. Al-Abbasi, Eman A. Alghamdi, Mohammed A. Baghdadi, Abdulmohsin J. Alamoudi, Ali M. El-Halawany, Hany M. El-Bassossy, Ali H. Aseeri and Ahmed M. Al-Abd

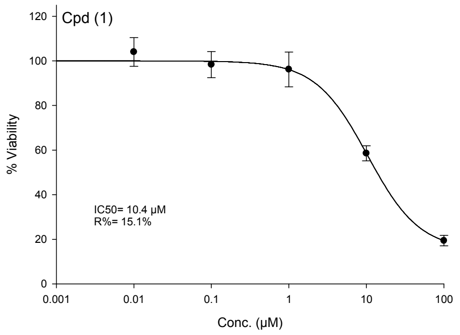

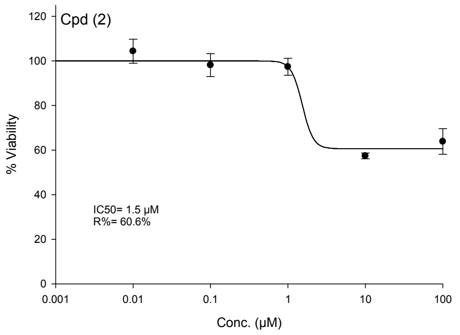

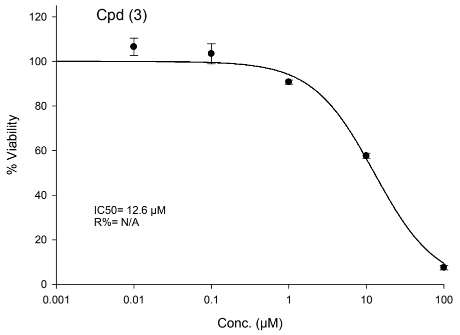

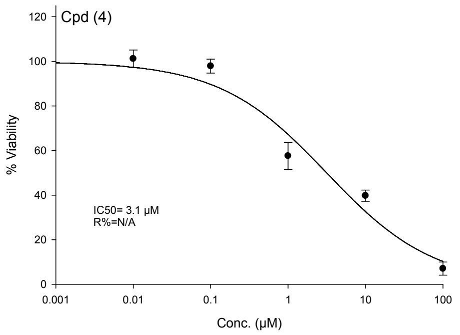

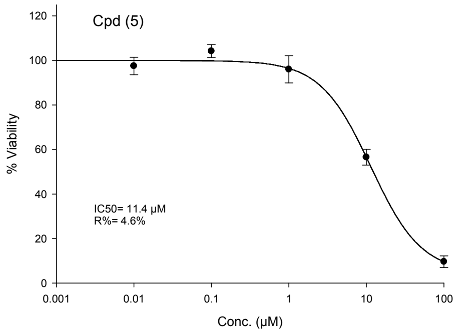

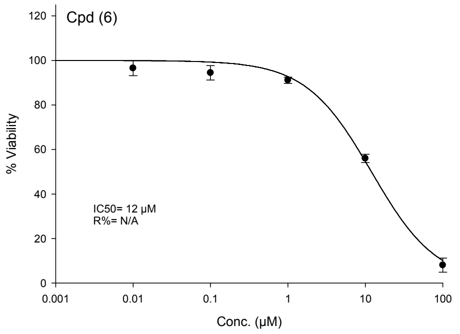

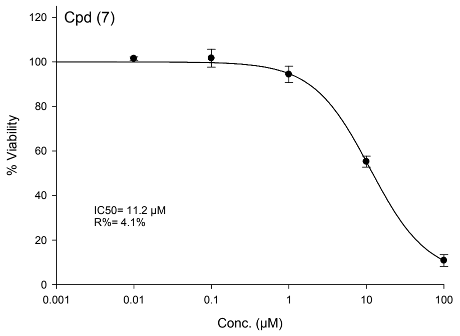

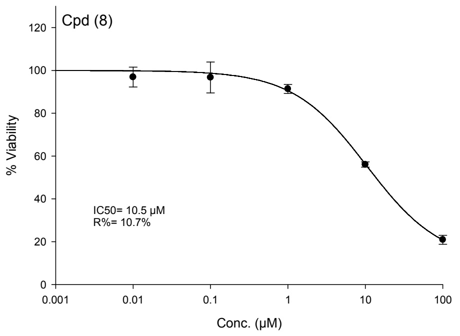

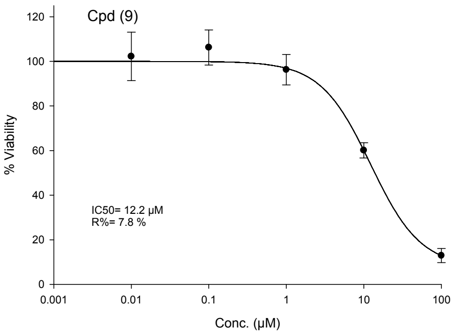

Figure S1. Dose response curves of different naturally occurring hydroxyphenylalkanes and diarylheptanoids against HCT-116 cells.

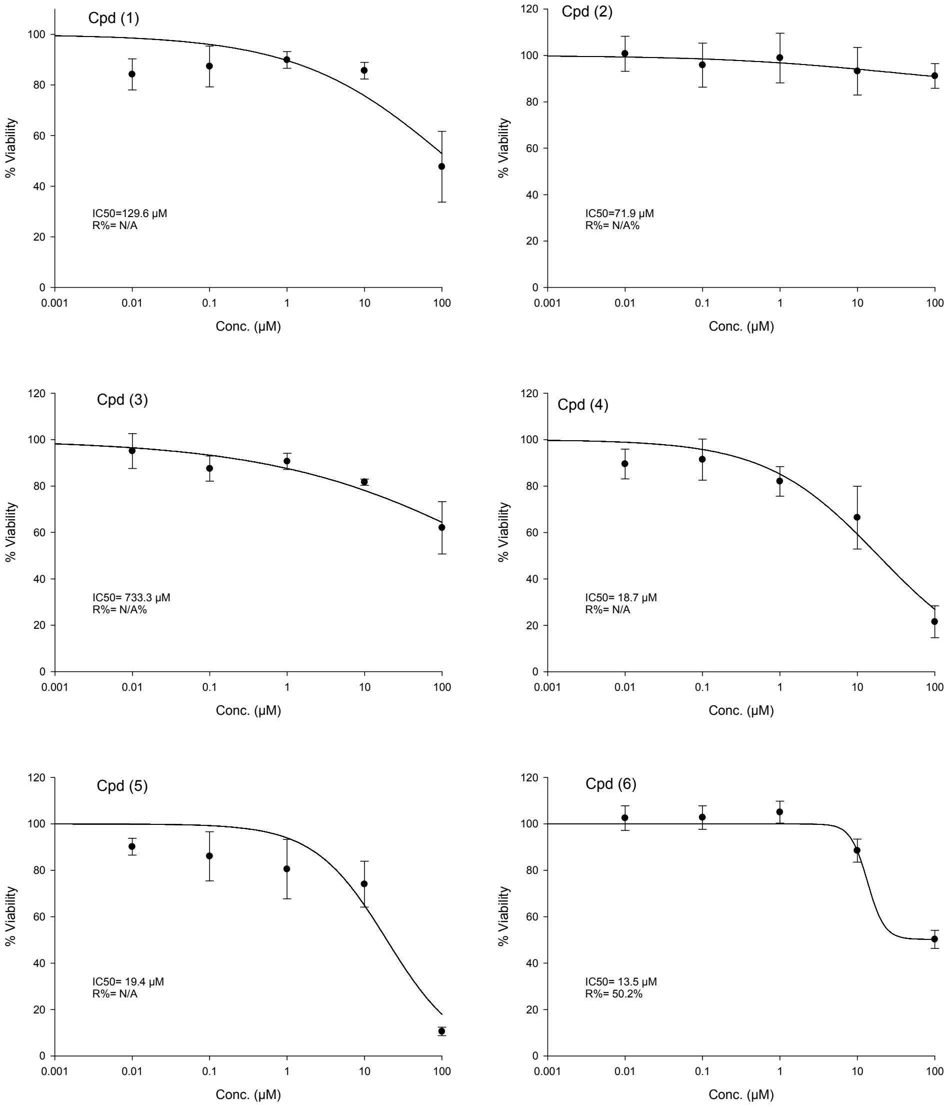

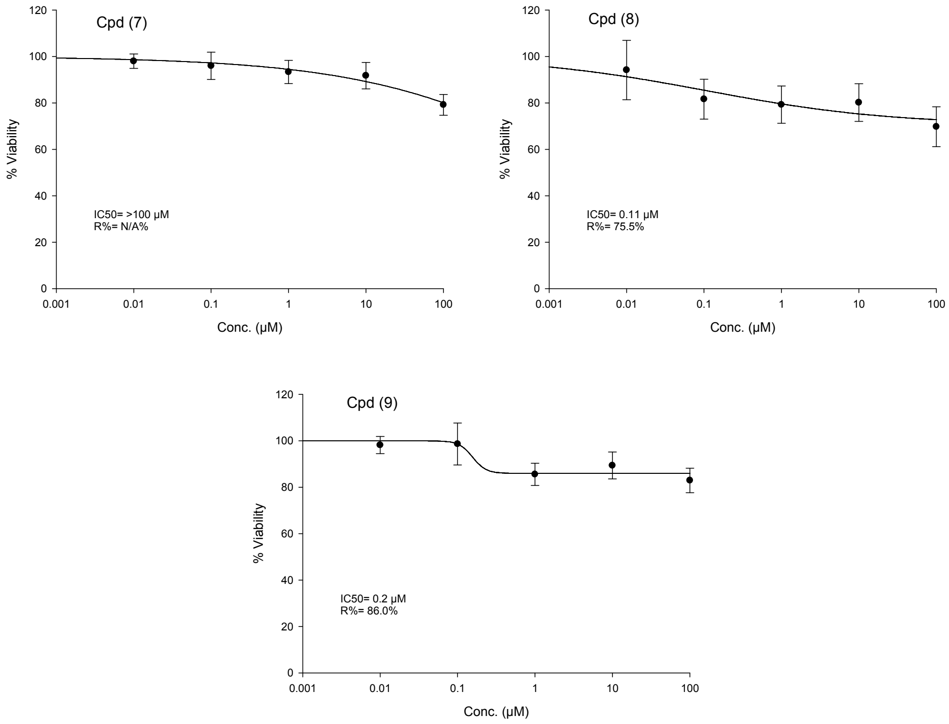

Figure S2. Dose response curves of different naturally occurring hydroxyphenylalkanes and diarylheptanoids against $HepG_2$ cells

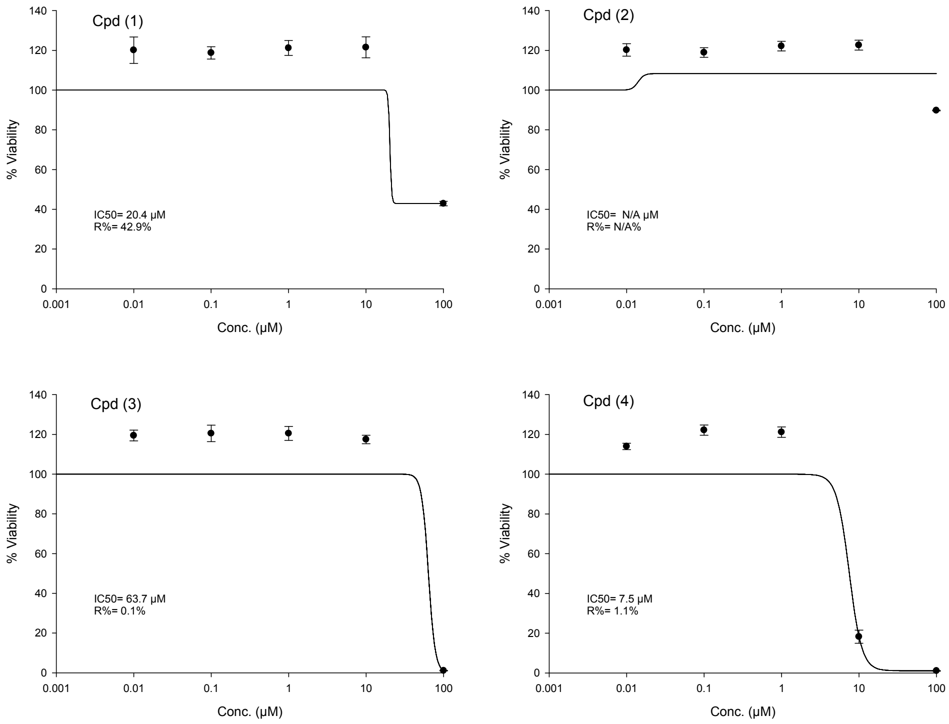

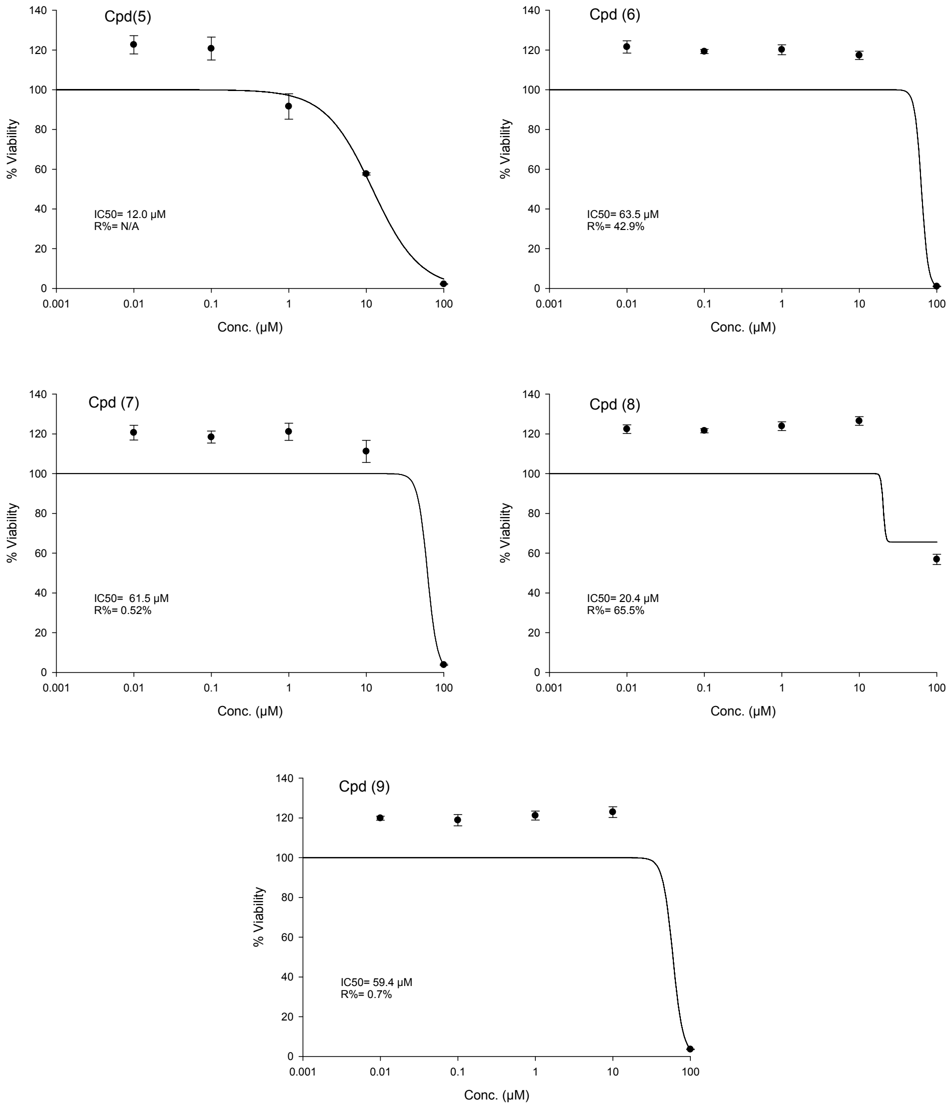

Figure S3. Dose response curves of different naturally occurring hydroxyphenylalkanes and diarylheptanoids against MCF-7 cells.

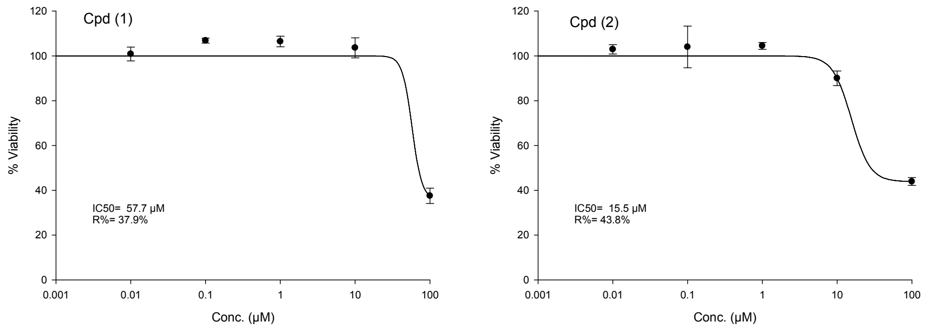

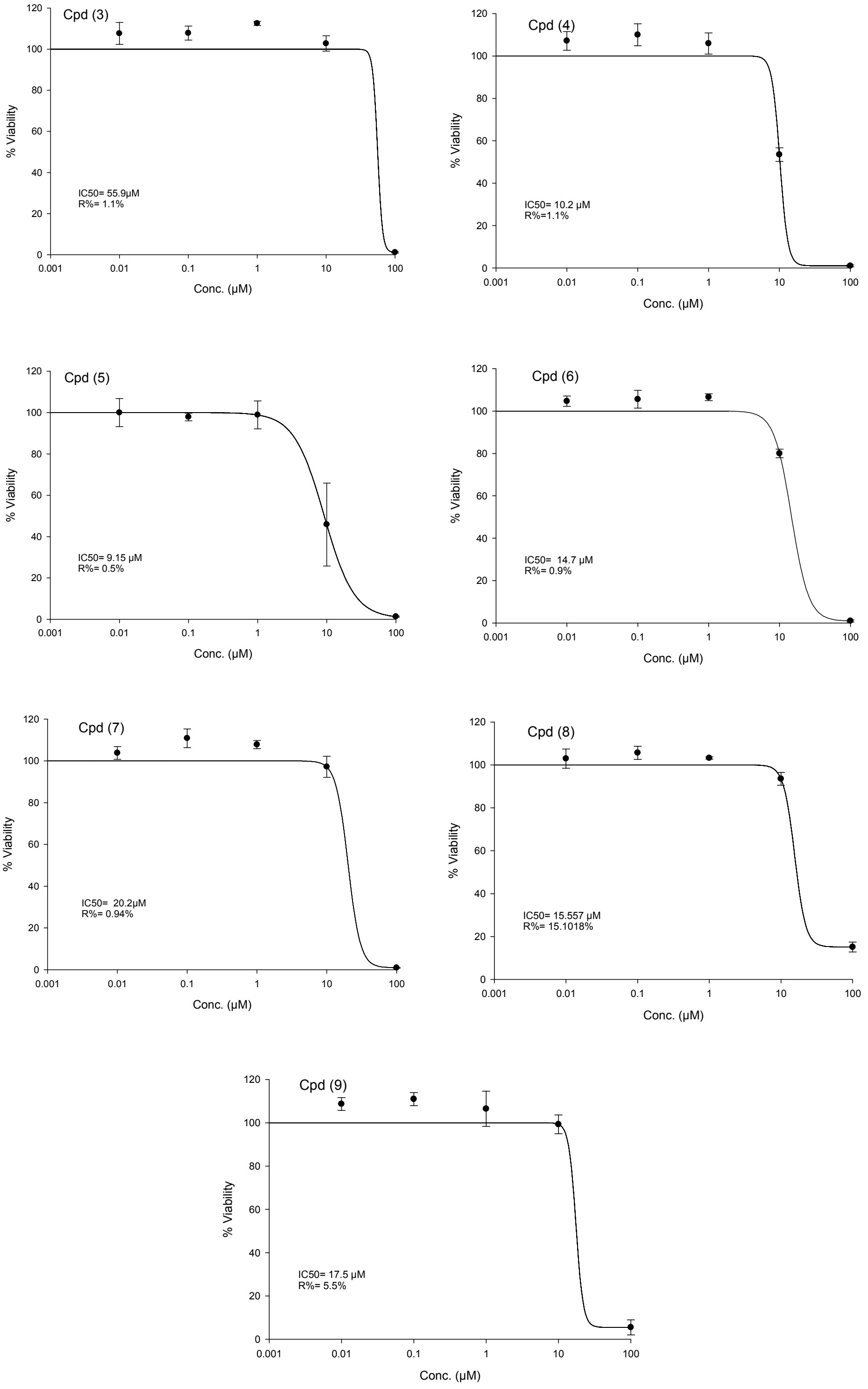

Figure S4. Dose response curves of different naturally occurring hydroxyphenylalkanes and diarylheptanoids against HeLa cells.

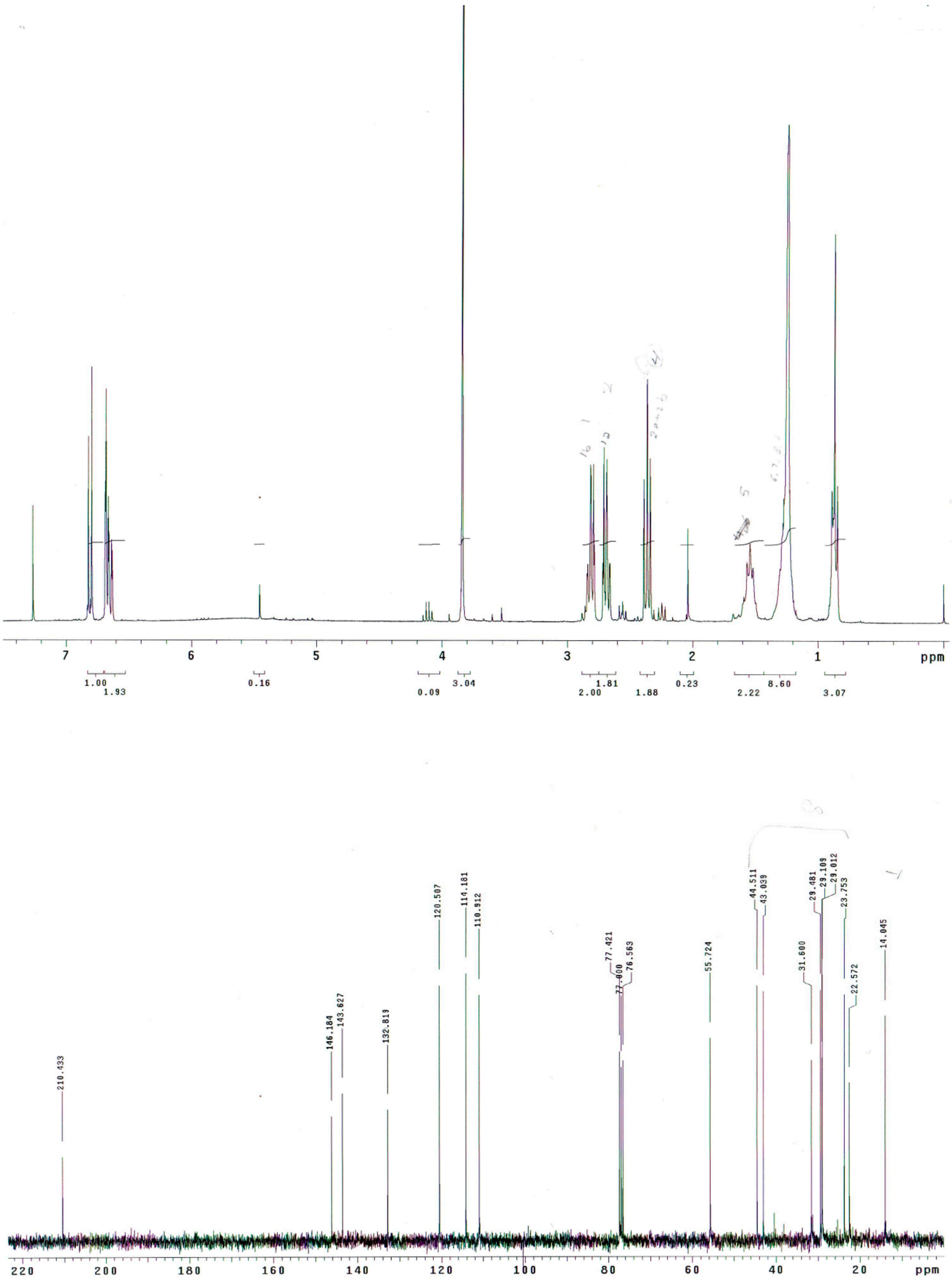

Figure S5. ^{1}H and ^{13}C-NMR charts of **6-paradol** (**1**).

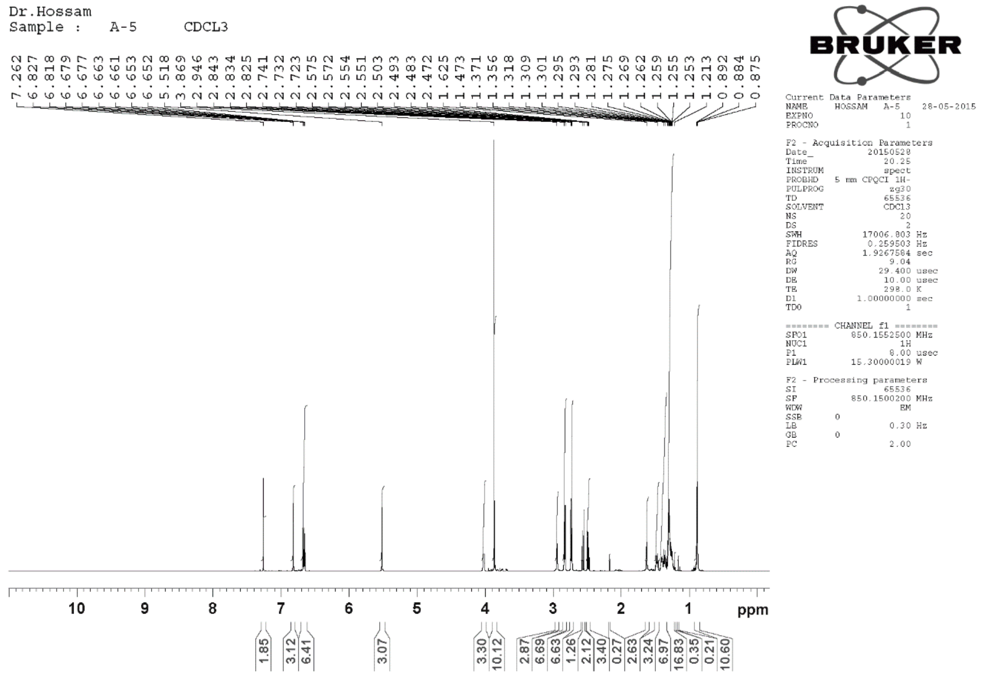

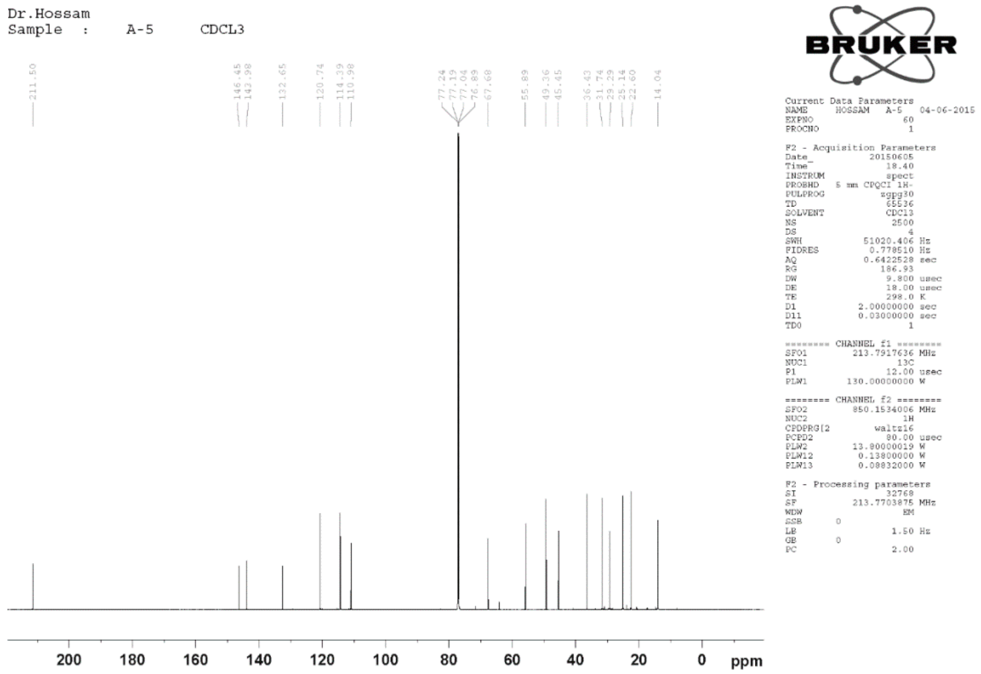

Figure S6. ^{1}H and ^{13}C-NMR charts of **6-gingerol** (**2**) (Samples were recorded on DRX-850 spectrometer (Bruker BioSpin, Billerica, MA, USA).

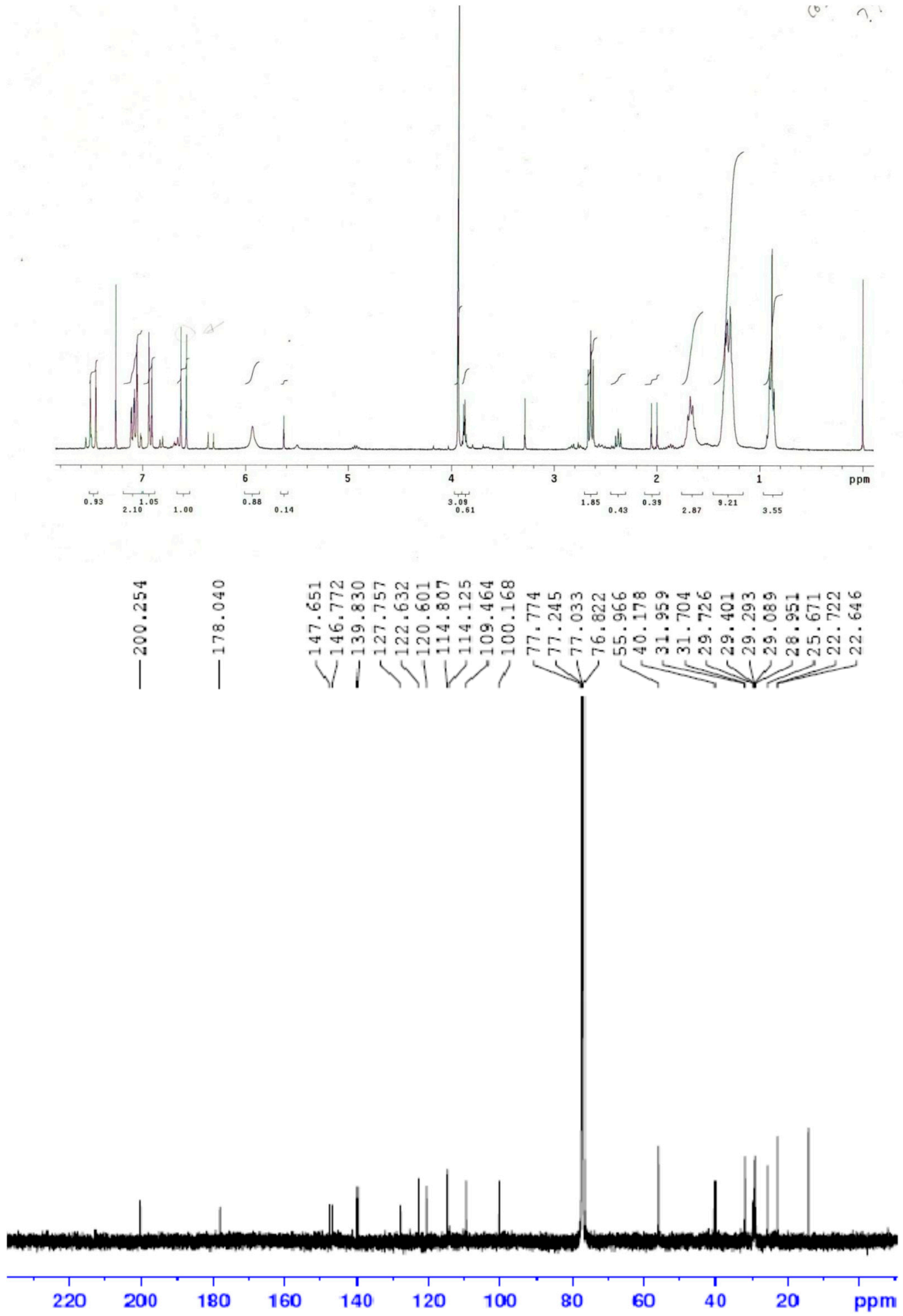

Figure S7. ^{1}H and ^{13}C-NMR charts of **dehydrogingerdione** (**3**).

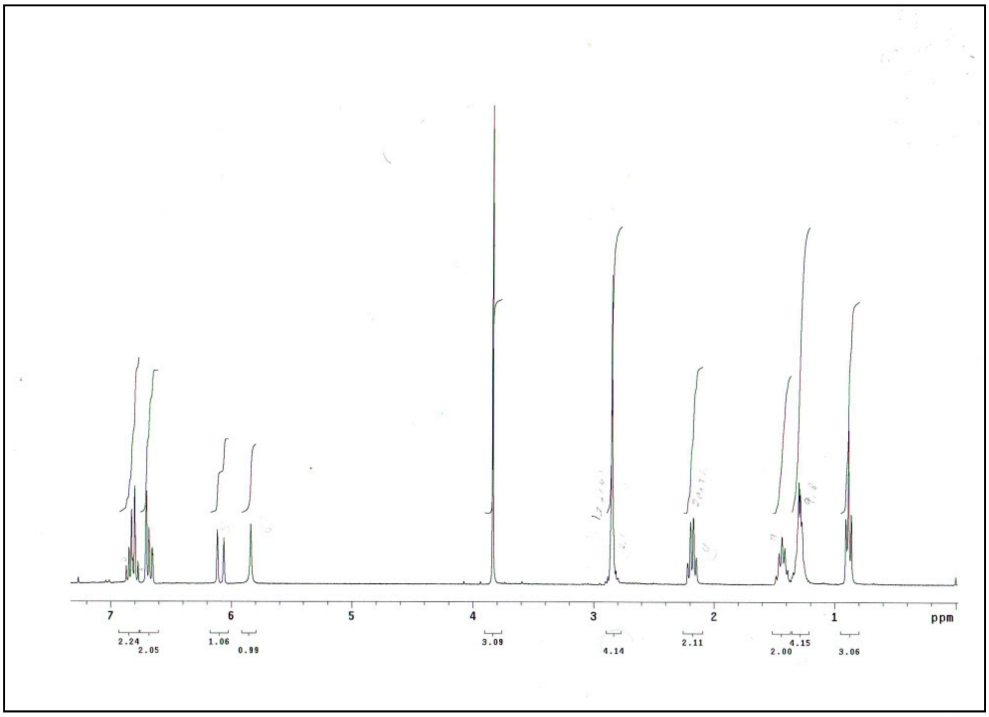

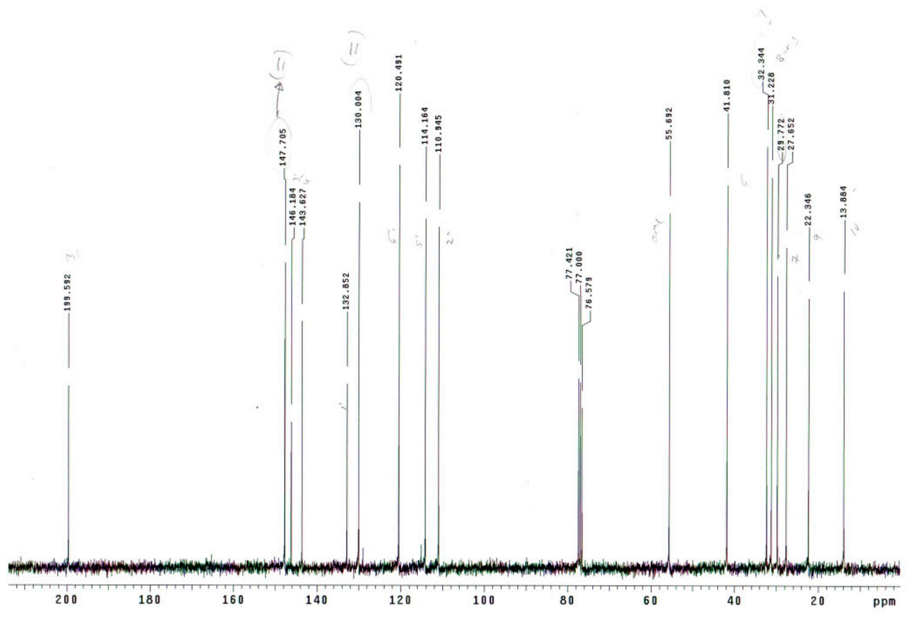

Figure S8. ^{1}H and ^{13}C-NMR charts of **6-shogoal** (**4**).

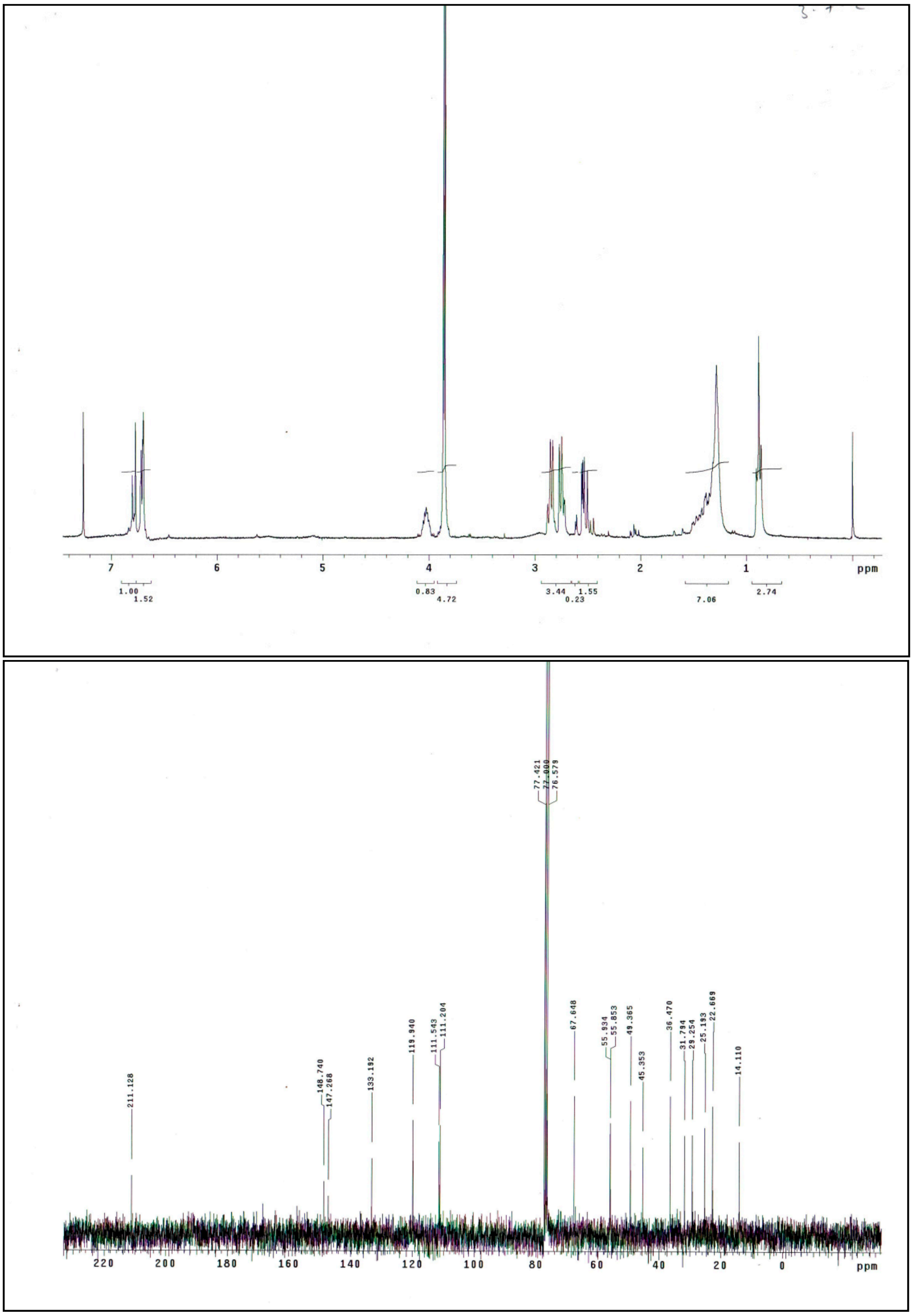

Figure S9. ^{1}H and ^{13}C-NMR charts of **4′-methoxy-6-gingerol** (**5**).

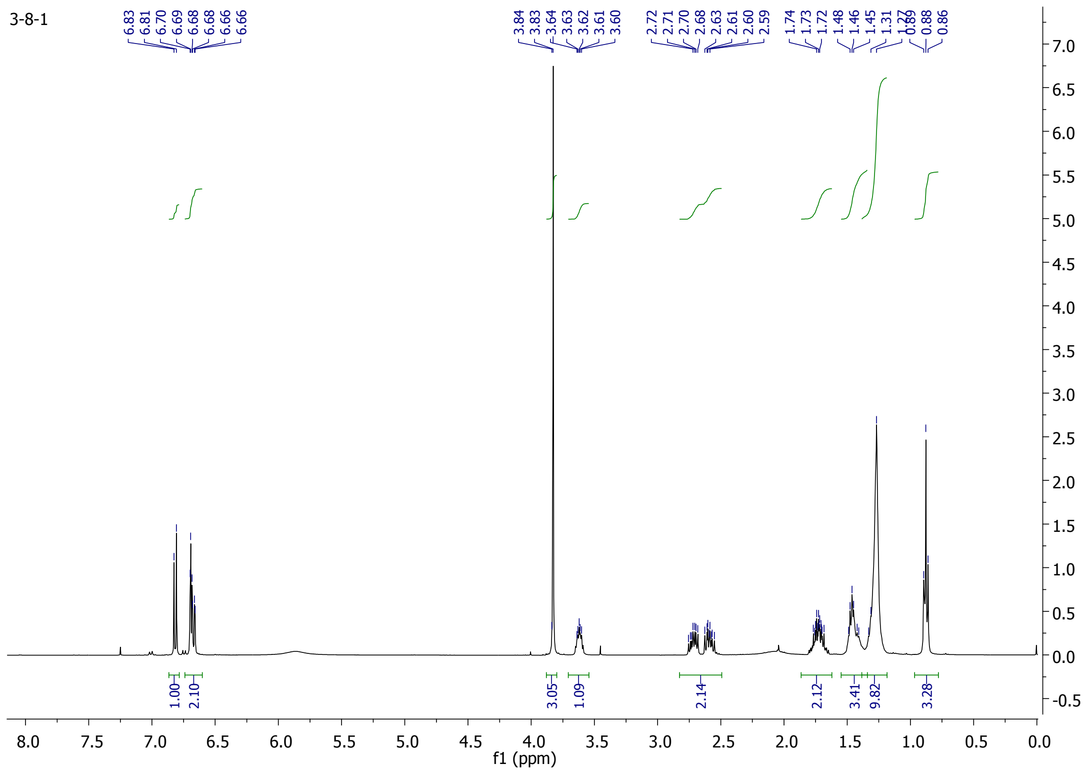

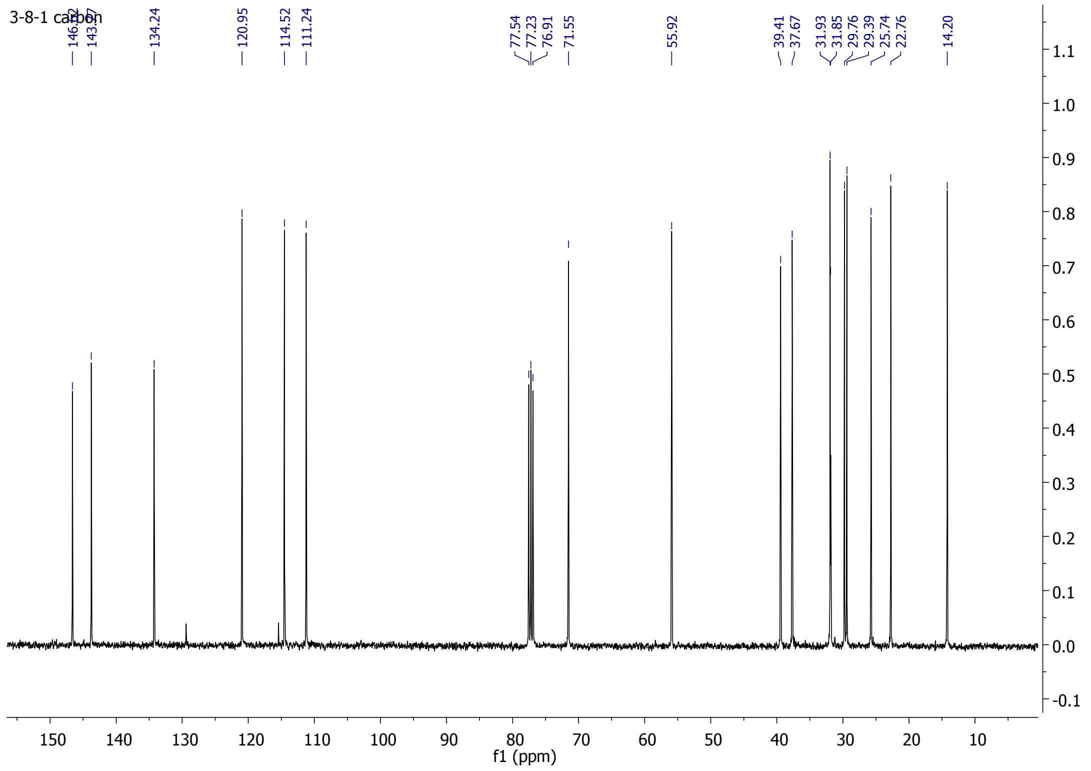

Figure S10. ^{1}H and ^{13}C-NMR charts of **dihydro-6-paradol** (**6**).

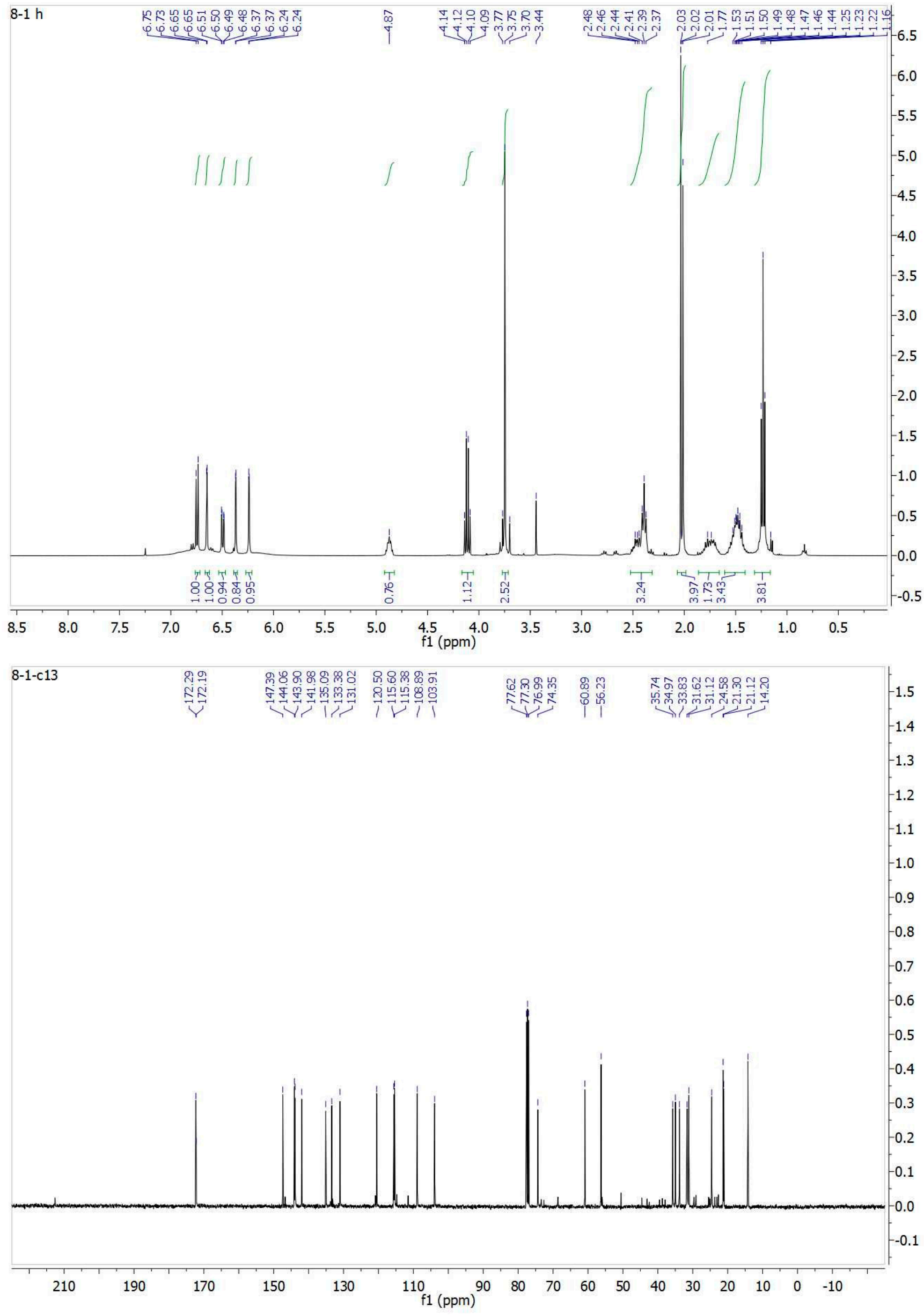

Figure S11. ^{1}H and ^{13}C-NMR charts of **DIACHEP** (**7**).

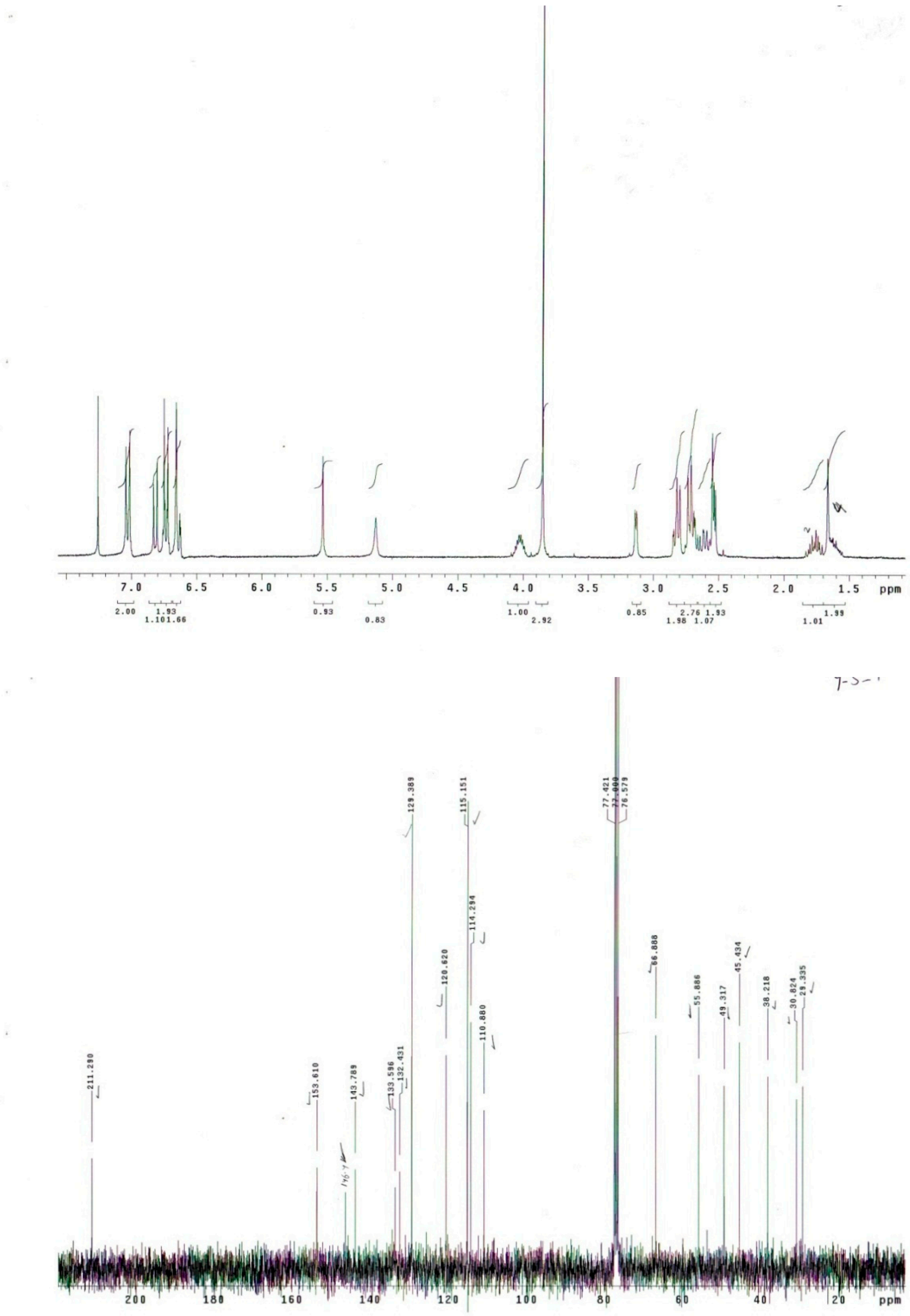

Figure S12. ^{1}H and ^{13}C-NMR charts of **dihydrogingerenone-C** (**8**).

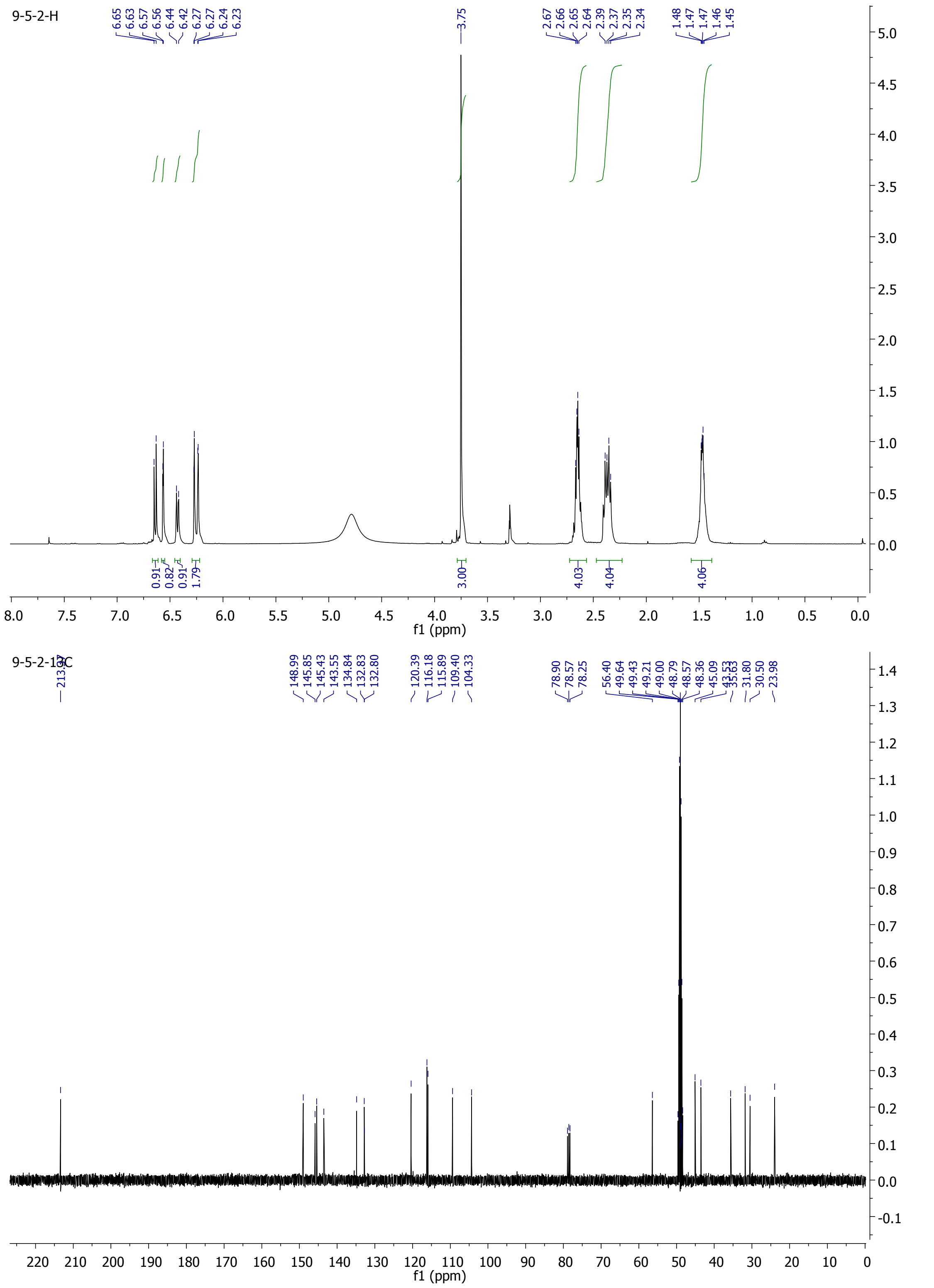

Figure S13. ^{1}H and ^{13}C-NMR charts of **dihydrogingerenone-A** (**9**).
